# γ-Aminobutyric acid type A receptor β1 subunit gene polymorphisms are associated with the sedative and amnesic effects of midazolam

**DOI:** 10.1186/s13041-024-01141-2

**Published:** 2024-09-27

**Authors:** Yoshihiko Kosaki, Daisuke Nishizawa, Junko Hasegawa, Kaori Yoshida, Kazutaka Ikeda, Tatsuya Ichinohe

**Affiliations:** 1 Department of Dental Anesthesiology, Tokyo Dental College, 2-9-18 Kanda- Misakicho, Chiyoda-Ku, Tokyo, 101-0061 Japan; 2 Department of Psychiatry and Behavioral Sciences, Addictive Substance Project, Tokyo Metropolitan Institute of Medical Science, 2-1-6 Kamikitazawa, Setagaya-ku, Tokyo, 156-8506 Japan; 3 Department of Neuropsychopharmacology, National Center of Neurology and Psychiatry, 4-1-1 Ogawahigashi-cho, Kodaira, Tokyo, 187-8553 Japan

**Keywords:** Benzodiazepine, GABA_A_ receptor, Intravenous sedation, Midazolam, Pharmacogenomics

## Abstract

Midazolam is widely used for intravenous sedation. However, wide interindividual variability is seen in the sensitivity to midazolam. The association between genetic factors and interindividual differences in midazolam sensitivity remains unclear. The present study explored the association between common genetic variants and sedative and amnesic effects of midazolam. This prospective study included patients who were scheduled to undergo dental procedures under intravenous sedation. The sedative effect was evaluated using the Ramsay sedation scale 5 min after midazolam (0.05 mg/kg) administration. We employed two parallel approaches in this study: genome-wide approach and candidate gene approach. The γ-aminobutyric acid type A receptor subunit genes were selected as candidate genes. Multivariate linear regression analyses were performed to investigate the association between the Ramsay sedation scale and genetic variants. We also analyzed the association between the presence of anterograde amnesia and genetic variants using multivariate binominal logistic regression analyses. The analyses were adjusted for potential confounding factors. A total of 191 patients were included in the analyses. In the genome-wide association analyses, no significant association was found between the genetic variants and Ramsay scores. In the candidate gene analyses, the rs73247636 (dominant model: *β* = 0.72 [95% confidence interval, 0.34 to 1.10], *P* < 0.001) and rs56278524 (dominant model: *β* = 0.73 [0.37 to 1.10], *P* < 0.001) polymorphisms of the *GABRB1* gene were significantly associated with Ramsay scores. Additionally, the rs73247636 (dominant model: odds ratio [OR] = 8.39 [2.36 to 29.85], *P* = 0.001) and rs56278524 (dominant model: OR = 15.26 [3.42 to 68.07], *P* < 0.001) polymorphisms were also significantly associated with the presence of anterograde amnesia. The rs73247636 and rs56278524 single-nucleotide polymorphisms of *GABRB1* were associated with the sedative and amnesic effects of midazolam.

**Supplementary Information:**

The online version contains supplementary material available at 10.1186/s13041-024-01141-2.

## Introduction

Midazolam is widely used for sedation in diagnostic and therapeutic procedures during the perioperative period [1]. In dentistry, intravenous sedation is useful for managing patients with dental phobia and a gagging problem, and midazolam is the most commonly used sedative for dental sedation [2, 3]. Dental procedural sedation has inherent risks because the airway is shared by the anesthesiologist and dentist [2]. Airway obstruction is one of the common causes of adverse outcomes [4, 5]. Midazolam also depresses the swallowing reflex and increases the potential risk for aspiration. Preventing airway complications and maintaining appropriate levels of sedation are critical for providing safe and effective dental sedation. However, wide interindividual variability is seen in the sensitivity to midazolam [6]. Midazolam should be individually titrated to the desired level of sedation.

Recently, numerous genetic studies have been conducted in the field of anesthesiology using various approaches. Several studies have investigated the effects of *CYP3A4* and *CYP3A5* gene polymorphisms on the pharmacokinetic properties of midazolam [7–9], whereas the effects of these polymorphisms remain controversial. Other studies reported that genetic polymorphisms were associated with the sedative effect of midazolam [10, 11]. Limited evidence is available on whether genetic variants affect the pharmacodynamics of midazolam compared with the pharmacokinetics of midazolam. One molecular target of the pharmacological effect of midazolam is the γ-aminobutyric acid type A (GABA_A_) receptor. The effects of diazepam are mediated by α subunits of GABA_A_ receptors [12], suggesting that the effects of midazolam should also be mediated by the same receptor subunits. However, the genetic contribution of GABA_A_ receptor subunits to the pharmacological effects of midazolam remains unknown [13]. Furthermore, no previous study has explored genome-wide associations with the pharmacokinetics and pharmacodynamics of midazolam. Only a few single-nucleotide polymorphisms (SNPs) of several genes have been investigated to date. Little is known about which genes contribute to interindividual differences in the effects of midazolam and the ways in which genetic factors affect physiological functions. Therefore, we conducted a candidate gene and genome-wide association study using DNA microarrays.

In the present study, we focused on the pharmacodynamics of midazolam rather its pharmacokinetics. We investigated the clinical response to midazolam during the sedation induction period with midazolam. We hypothesized that common genetic variants rather than low-frequency variants would affect interindividual differences in the sedative and amnesic effects of midazolam. The primary aim of this study was to investigate whether genetic variants, especially variants of GABA_A_ receptor subunit genes, are associated with the sedative effect of midazolam. The secondary aim of this study was to evaluate whether these variants were also associated with the amnesic effect of midazolam if genetic associations with sedative effects were observed in the primary study.

## Materials and methods

This exploratory prospective study was approved by the Tokyo Dental College Ethics Committee (Tokyo, Japan; approval No. 919; February 13, 2019) and Tokyo Metropolitan Institute of Medical Science Ethics Committee (Tokyo, Japan; approval No. 18–46; March 7, 2019). Patients were recruited at Tokyo Dental College Suidobashi Hospital, Tokyo, Japan, between April 2019 and March 2020. Genetic analyses were conducted at the Tokyo Metropolitan Institute of Medical Science, Tokyo, Japan. All of the patients provided written informed consent before participating in this study.

Patients who were scheduled to undergo dental procedures under intravenous sedation were eligible for this study. The inclusion criterion was age between 20 and 60 year. The exclusion criteria were (1) American Society of Anesthesiologists (ASA) physical status III or higher, (2) known allergy to midazolam, (3) psychotropic drug use, including benzodiazepine use, within the past 3 months, and (4) non-Japanese descent.

### Sedation management

No premedication was administered. The patients were seated in a dental chair in the semi-supine position with the head up at a 40-degree angle. Vital signs, including noninvasive blood pressure, pulse rate, and oxygen saturation (measured by pulse oximetry [SpO_2_]), and Bispectral Index (BIS) values were monitored. A BIS electrode was applied on the patient’s forehead. BIS values were continuously recorded with a BIS monitor (Covidien, Tokyo, Japan). The Ramsay sedation scale was used to evaluate the patients’ level of sedation: score of 1, patient anxious and agitated or restless or both; 2, patient cooperative, oriented and tranquil; 3, patient drowsy, but responds to commands; 4, asleep, brisk response; 5, asleep, sluggish response; 6, no response [14]. Patients with no clinical response to midazolam were deemed to have a Ramsay score of 1. To evaluate the amnesic effect of midazolam, the patients were requested to memorize a word and the region where we would collect buccal swab samples during sedation.

After recording baseline vital signs and determining the BIS value, midazolam 0.05 mg kg^− 1^ (Teva Takeda Pharma, Aichi, Japan) was administered intravenously in approximately 1 min. Five minutes after initial midazolam administration, the Ramsay score was recorded by a dentist anesthesiologist (Y. K.) in all cases. The same variables as baseline were also recorded. If SpO_2_ was less than 90%, then a jaw thrust maneuver was performed to maintain the patient’s airway, and supplemental oxygen was supplied *via* a nasal cannula. Any adverse events, including airway obstruction, desaturation, apnea, and unstable hemodynamic, were recorded.

Buccal swab sampling and saying a word occurred immediately after Ramsay score recording. Buccal swab samples were collected for genotyping from either left or right buccal mucosa, and the patients were told a word twice by the dentist anesthesiologist (Y. K.). The word was selected in a randomized manner from the following words (in Japanese): apple, banana, grape, orange, and peach. After data collection, the dental procedure began. The level of sedation was adjusted by the attending dentist anesthesiologist using midazolam with or without propofol. After finishing the procedure, the patients were moved to the recovery room. Amnesic effects, namely the memories of buccal swab sampling and saying a word, were assessed 30 min after confirming that the patients were near their baseline level of consciousness. The patients were considered to have anterograde amnesia if they recalled neither the word nor the left or right side where the buccal swab samples were collected.

### DNA genotyping

Total genomic DNA was extracted from buccal mucosa samples using the QIAamp DNA Micro kit (Qiagen, Hamburg, Germany) according to the manufacturer’s instructions. The extracted DNA was stored at 4 °C until analysis. After all of the clinical data were collected, genotyping was performed on an Infinium Asian Screening Array-24 v1.0 BeadChip (Illumina, San Diego, California, USA) according to the manufacturer’s instructions. Genotypes were called using GenomeStudio v2.0.5 with the Genotyping v2.0.5 module (Illumina). Quality control was performed for the DNA samples and genetic markers. Samples were excluded if the sample call rate was less than 97%. Genetic markers were excluded if each quality metric did not meet the hard cutoff thresholds that are recommended by Illumina, including call frequency < 97%, Cluster Sep ≤ 0.3, AA R Mean ≤ 0.2, AB R Mean ≤ 0.2, and BB R Mean ≤ 0.2. The other multi-variable metrics were higher or lower than the hard cutoff values. In addition to the quality control criteria, variants with a minor allele frequency less than 5% (i.e., rare and low-frequency variants) were filtered out from statistical analyses.

### Sample size

Although no data on genome-wide genetic variants or the sedative effect of midazolam were available, we performed a priori power analysis using Quanto 1.2.4 [15] based on Cohen’s small, medium, and large *R*^2^ effect sizes of 0.02, 0.13, and 0.26, respectively [16]. The type I error rate, adjusted for multiple testing, was set at 7.6 × 10^− 8^ (0.05/660,000) because the Infinium Asian Screening Array-24 v1.0 BeadChip contains approximately 660,000 markers. Based on a two-sided α error rate of 7.6 × 10^− 8^ and statistical power of 0.8, the sample sizes were calculated as 1,914 for a small effect size, 278 for a medium effect size, and 128 for a large effect size. We sought to recruit at least 128 patients during the 1-yr exploratory study period to examine strong genetic associations with the sedative effect of midazolam.

### Statistical analysis

We used two parallel approaches in this study. First, we tested all genetic variants using the genome-wide approach. Second, we tested variants of genes that are related to midazolam sensitivity using the candidate gene approach. For the candidate gene analyses, we focused on GABA_A_ receptors. Although there are 19 GABA_A_ receptor subunits (α1–6, β1–3, γ1–3, δ, ε, θ, π, and ρ1–3), the α4, α6, π, and ρ subunits are insensitive to classic 1,4-benzodiazepines [17–21]. Thus, we chose genes that encode the α1, 2, 3, 5, β1–3, γ1–3, δ, ε, and θ subunits in humans for candidate gene analyses. We selected 372 SNPs of 13 genes (*GABRA1*, *GABRA2*, *GABRA3*, *GABRA5*, *GABRB1*, *GABRB2*, *GABRB3*, *GABRG1*, *GABRG2*, *GABRG3*, *GABRD*, *GABRE*, and *GABRQ*) as the candidate SNPs.

Multivariate linear regression analyses were conducted to investigate the association between genotypes and Ramsay sedation scores as an index of the sedative effect of midazolam. Ramsay scores were used as numeric phenotype values for the dependent variable. Genotypes were used for independent variables. Age, sex, and body mass index (BMI) were included as covariates to adjust potential confounding factors in the linear regression models. Additive, dominant, and recessive genetic models were used for the analyses. Genetic variants on the X, Y, and mitochondrial chromosomes (except for the pseudoautosomal region) were excluded from the analyses of the dominant and recessive models. Values of *P* were adjusted for multiple testing with false discovery rate correction (*Q* value) [22]. Statistical significance was set at a *Q* value less than 0.05. Deviations from Hardy-Weinberg equilibrium were tested using Fisher’s exact test at a type I error rate of 0.001.

Additional analyses were performed for genetic variants that had a *Q* value less than 0.05 in the genome-wide association analyses and candidate gene analyses. To explore the association between the amnesic effect of midazolam and genetic variants, multivariate binominal logistic regression analyses were conducted using the covariates age, sex, and BMI. The presence of anterograde amnesia was used as the phenotype value for the dependent variable. Genotypes were used for independent variables. The genetic models that were used for the analyses were the same as the linear regression analyses. Statistical significance was set at a *P* value less than 0.05 after correcting for multiple testing using Bonferroni adjustment.

All of the statistical analyses were performed using PLINK 1.90 beta 6.18 [23, 24]. All of the tests were two-tailed. According to the PLINK sample information file format, the presence of anterograde amnesia was coded as the following: 1 (patients with recall [‘control’]) and 2 (patients with amnesia [‘case’]). Male and female were also coded as 1 and 2, respectively. Continuous variables are presented as means (standard deviation) or medians (interquartile range). Categorical variables are described as numbers with percentages. Effect size estimations are reported as regression coefficient (*β*) or odds ratio (OR) with 95% confidence intervals (CIs).

## Results

**Fig. 1:**
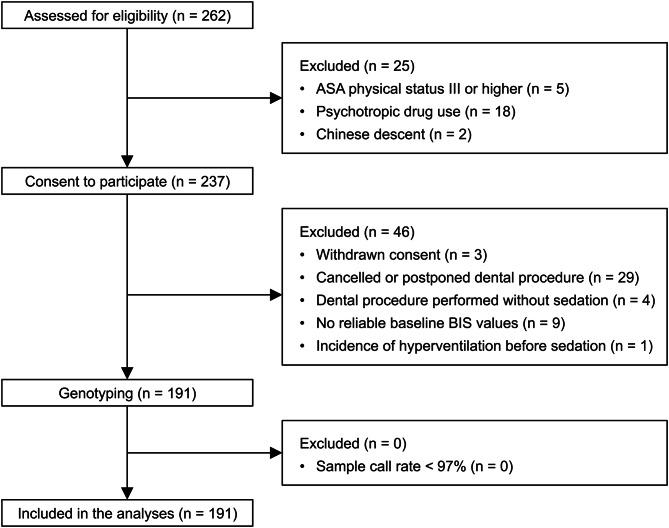
Flowchart of the present study. ASA, American Society of Anesthesiologists; BIS, Bispectral index

The flowchart of this study is presented in Fig. 1. Of the 262 patients who were recruited, 191 were included in the final analyses. Demographic and intraoperative data of the remaining 191 patients are summarized in Table 1. The median Ramsay score 5 min after the initial midazolam administration was 3 (interquartile range, 2 to 4). Anterograde amnesia occurred in 132 patients (69%). Adverse events after the initial midazolam administration occurred in 25 patients (13%) for airway obstruction and four patients (2%) for transient apnea. Of these 29 patients, the median Ramsay score was 4 (interquartile range, 4 to 5). The mean age was 41 years (range, 22 to 58 years). The mean BMI was 24.5 (range, 18.7 to 33.8), and there were 21 males. Although 14 of these 29 patients (7%) resulted in desaturation (SpO_2_ less than 90%), all of them recovered immediately after the airway maneuver, being supplied with supplemental oxygen and/or taking deep breaths. All of the DNA samples were successfully genotyped at a call rate of more than 97%. The mean sample call rate was 99.46% (range, 98.90–99.81%). Of the 659,184 markers on the microarray, 651,087 met the quality control criteria. After minor allele frequency filtering, 314,148 SNPs and 256 insertion–deletions remained.

**Table 1:** Patient characteristics

Age, yr (range)	37 (20‒60)
Sex, male/female	77/114
Height, cm	164 ± 9
Weight, kg	59 ± 12
BMI, kg m^− 2^	21.8 ± 3.6
ASA physical status, I/II	118/73
Type of dental procedure, n (%)	
Oral surgery	153 (80)
Dental restoration	33 (17)
Prosthodontics	5 (3)
Indications for sedation, n (%)	
Deeply impacted third molar extraction	69 (36)
Dental phobia	67 (35)
Gagging problem	35 (18)
History of vasovagal reflex	11 (6)
Other	9 (5)
Duration of procedure, min	33 ± 21
Duration of sedation, min	47 ± 21
Additional midazolam use, n (%)	93 (49)
Total midazolam dose, mg	3.5 ± 0.9
Propofol use, n (%)	121 (63)
Total propofol dose, mg	106 ± 82
MAP, mm Hg	
Baseline	92.3 ± 15.3
After midazolam dose	84.0 ± 12.2
Pulse rate, beats min^− 1^	
Baseline	79.4 ± 14.5
After midazolam dose	82.4 ± 13.1
SpO_2_, %	
Baseline	98.5 ± 1.3
After midazolam dose	96.6 ± 2.0
BIS value	
Baseline	96.3 ± 2.8
After midazolam dose	76.7 ± 8.1
Ramsay sedation scale, n (%)	
Score 1	14 (7)
Score 2	43 (22)
Score 3	57 (30)
Score 4	51 (27)
Score 5	23 (12)
Score 6	3 (2)
Presence of amnesia, n (%)	132 (69)
Adverse events, n (%)	
Airway obstruction	25 (13)
Transient apnea	4 (2)
Desaturation	14 (7)

The data are expressed as mean ± SD or number (%). The Ramsay sedation score was recorded 5 min after the initial midazolam administration. BMI, body mass index; ASA, American Society of Anesthesiologists; MAP, mean arterial pressure; BIS, Bispectral index

### Genome-wide association analyses

A total of 314,148 SNPs and 256 insertion–deletions were used for the linear regression analysis in the additive model. Among them, 14,647 SNPs and 4 insertion–deletions on the X, Y, and mitochondrial chromosomes were excluded from the dominant and recessive models. A total of 299,501 SNPs and 252 insertion–deletions were used for the analyses using the dominant and recessive models.

**Fig. 2:**
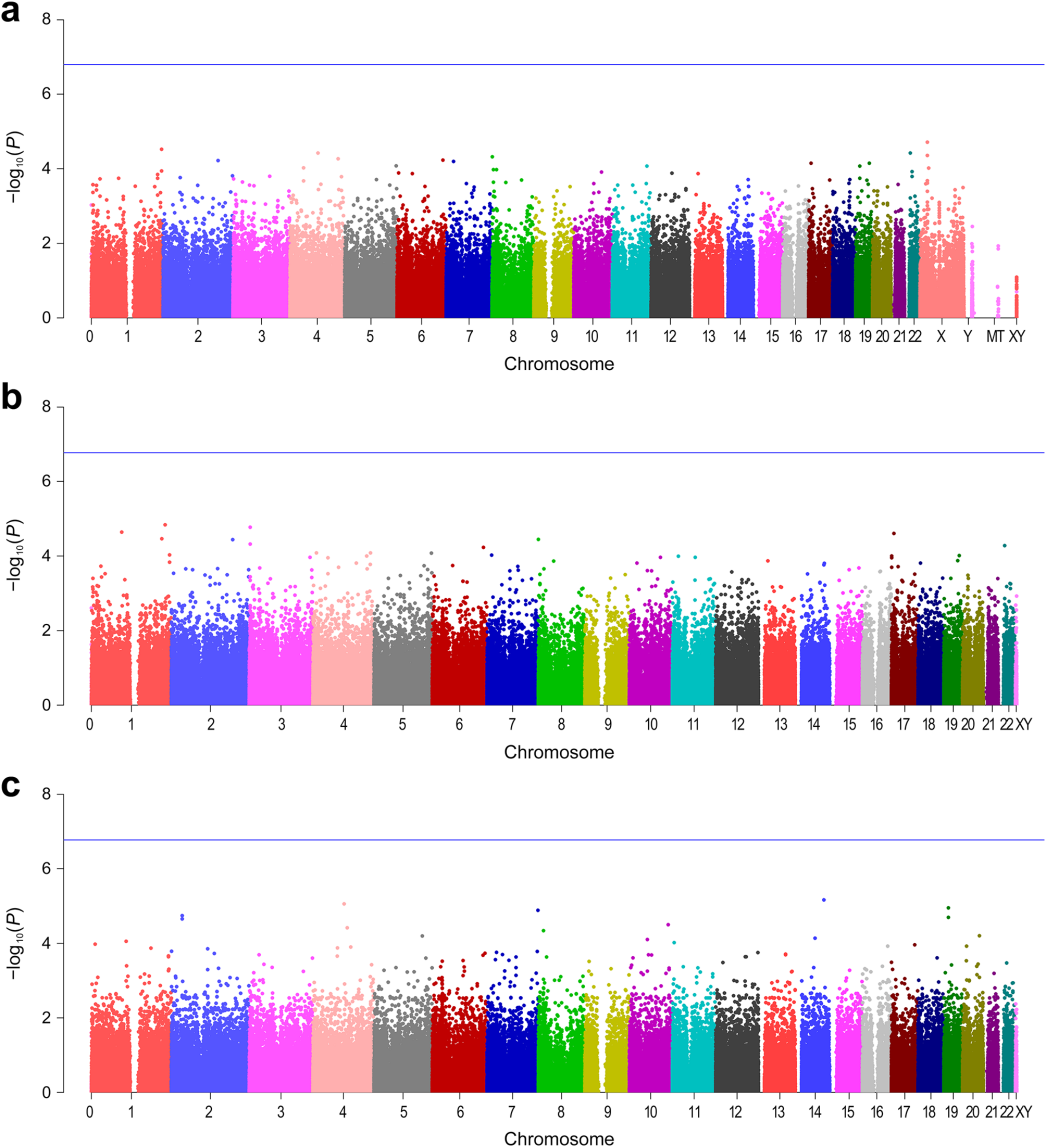
Manhattan plots of the genome-wide association analyses of the Ramsay sedation score in (**a**) the additive model, (**b**) the dominant model and (**c**) the recessive model. The − log_10_(*P*) values are plotted for all variants across the chromosomes. The blue lines indicate the genome-wide significance threshold (*P* = 0.05/314,404 [1.59 × 10^− 7^] in the additive model; *P* = 0.05/299,753 [1.67 × 10^− 7^] in the dominant and recessive models). Chromosome 0 indicates unmapped or multiple mapped single-nucleotide polymorphisms based on Genome Reference Consortium Human Build 38. XY, pseudoautosomal region; MT, mitochondrial DNA

None of the SNPs and insertion–deletions were significantly associated with Ramsay sedation scores in the genetic models (Fig. 2). All of the genetic variants with *P* values less than 0.0001 are listed in Table S1 (Additional file 1). The quantile-quantile plots did not show large deviations from the null hypothesis of a uniform distribution, suggesting minimal confounding effects from population stratification (Fig. S1, Additional file 2). Of the genetic markers that were analyzed, rs9323838 SNP on chromosome 14 had the strongest association with Ramsay sedation scores in the recessive model (*β* = − 0.95 [95% CI, − 1.36 to − 0.55], nominal *P* = 6.8 × 10^− 6^), although the association was not statistically significant (*Q* = 0.855). Of the 314,148 SNPs, 261 on autosomal chromosomes and 300 on sex chromosomes showed deviation from Hardy-Weinberg equilibrium (*P* < 0.001; data not shown).

### Candidate gene analyses

Using the candidate gene approach, 372 SNPs of 13 genes were tested. Among these, two SNPs were significantly associated with Ramsay sedation scores in the additive and dominant models (Table 2; see Table S2, Additional file 3, for a list of all results from the candidate gene analyses): rs73247636 (additive model: *β* = 0.64 per G allele [95% CI, 0.31 to 0.97], *Q* = 0.039; dominant model: *β* = 0.72 [0.34 to 1.10], false discovery rate *P* = 0.047), and rs56278524 (additive model: *β* = 0.63 per G allele [0.32 to 0.93], *Q* = 0.035; dominant model: *β* = 0.73 [0.37 to 1.10], *Q* = 0.035), both of which were polymorphisms of *GABRB1*. Table S3 summarised regression coefficients of the covariates which were included in these regression models (Additional file 4). All the observed genotype frequencies of the candidate genes were in Hardy-Weinberg equilibrium (*P* > 0.001, Table S2, Additional file 3).

**Table 2:** Association between Ramsay sedation score and variants

							Additive Model				Dominant Model				Recessive Model			
						HWE												
Gene	Chr	SNP	Position	A1/A2	MAF	*P*	*β*	95% CI	*P*	*Q*	*β*	95% CI	*P*	*Q*	*β*	95% CI	*P*	*Q*
*GABRA1*	5	rs10042696	161,879,765	C/T	0.35	0.20	0.10	− 0.14 to 0.35	**0.408**	0.960	0.16	− 0.15 to 0.48	**0.309**	0.957	0.03	− 0.49 to 0.55	0.921	0.993
*GABRA2*	4	rs77166883	46,314,374	T/C	0.18	1	− 0.40	− 0.68 to − 0.12	**0.006**	0.347	− 0.47	− 0.80 to − 0.15	**0.004**	0.246	− 0.42	− 1.31 to 0.47	0.357	0.941
*GABRA3*	X	rs389292	152,372,982	C/T	0.18	1	− 0.26	− 0.58 to 0.06	**0.109**	0.763								
*GABRA5*	15	rs8028947	26,928,735	T/C	0.09	0.17	− 0.35	− 0.71 to 0.02	**0.065**	0.749	− 0.26	− 0.68 to 0.16	0.229	0.913	− 1.71	− 2.94 to − 0.49	**0.007**	0.933
*GABRB1*	4	rs73247636	47,092,489	A/G	0.10	0.13	0.64	0.31 to 0.97	2.1 × 10^− 4^	**0.039**	0.72	0.34 to 1.10	3.0 × 10^− 4^	**0.047**	1.03	− 0.05 to 2.11	0.064	0.933
		rs56278524	47,110,636	A/G	0.12	0.15	0.63	0.32 to 0.93	**9.4 × 10** ^**− 5**^	**0.035**	0.73	0.37 to 1.10	**1.1 × 10** ^**− 4**^	**0.035**	0.94	− 0.02 to 1.91	0.058	0.933
*GABRB2*	5	rs17521304	161,404,861	T/C	0.10	0.10	0.46	0.11 to 0.80	**0.010**	0.391	0.48	0.08 to 0.89	**0.020**	0.482	1.03	− 0.05 to 2.11	0.064	0.933
*GABRB3*	15	rs73368376	26,641,680	A/G	0.09	1	0.38	− 0.01 to 0.76	**0.057**	0.728	0.37	− 0.04 to 0.77	**0.077**	0.612	1.30	− 0.84 to 3.44	0.236	0.941
*GABRD*	1	rs28574670	2,027,822	G/A	0.24	0.24	− 0.08	− 0.32 to 0.17	**0.535**	0.960	− 0.04	− 0.35 to 0.28	0.816	0.974	− 0.32	− 0.91 to 0.27	**0.291**	0.941
*GABRE*	X	rs2266859	151,973,735	A/G	0.47	0.45	− 0.22	− 0.46 to 0.02	**0.068**	0.749								
*GABRG1*	4	rs1353640	46,104,826	A/C	0.33	0.20	0.07	− 0.16 to 0.30	0.539	0.960	0.17	− 0.14 to 0.49	**0.278**	0.957	− 0.08	− 0.55 to 0.38	0.732	0.993
*GABRG2*	5	rs11135176	162,095,550	C/T	0.30	0.61	− 0.24	− 0.47 to 0.00	**0.047**	0.677	− 0.27	− 0.58 to 0.04	0.086	0.633	− 0.40	− 0.91 to 0.12	0.132	0.933
*GABRG3*	15	rs7173587	27,219,705	C/T	0.09	0.37	− 0.63	− 1.03 to − 0.23	**0.002**	0.269	− 0.63	− 1.03 to − 0.23	**0.002**	0.201	NA	NA	NA	NA
*GABRQ*	X	rs10482208	152,642,426	C/T	0.11	1	0.08	− 0.31 to 0.47	**0.679**	0.963								

Variants with lowest *P* values in each gene and with false discovery rate *P* < 0.05 are listed. Variants on the X chromosome were excluded from the analyses for the dominant and recessive models. The regression coefficients (*β*) represent the effect size and direction with the major allele as the reference allele. Values in bold indicate the lowest *P* values within each gene and significant results with a false discovery rate *P* < 0.05

Chr, chromosome; SNP, single-nucleotide polymorphism; Position, chromosomal position in Genome Reference Consortium Human Build 38; A1, major allele; A2, minor allele; MAF, minor allele frequency; HWE, Hardy-Weinberg equilibrium exact test; *β*, regression coefficient; *Q*, false discovery rate *P*; NA, not available

### Additional analyses of rs73247636 and rs56278524

Only two SNPs, rs73247636 and rs56278524, had a *Q* value less than 0.05 in the genome-wide association analyses and candidate gene analyses (Table 2). The Bonferroni-corrected threshold was set at a *P* value less than 0.05/2. Both rs73247636 and rs56278524 were significantly associated with the presence of amnesia in the additive and dominant models (Table 3 and Table S4, Additional file 5). Anterograde amnesia occurred more often in carriers of the minor G allele of rs73247636 (OR, 8.39 [95% CI, 2.36 to 29.85]; adjusted *P* = 0.002) and rs56278524 (OR, 15.26 [3.42 to 68.07]; adjusted *P* < 0.001) compared with homozygous carriers of the major allele based on the dominant model. Genotypes and detailed information of the 29 patients who presented adverse effects of midazolam are shown in Table S5 (Additional file 6).

**Table 3:** Association between *GABRB1* polymorphisms and the amnesic effect of midazolam

		Additive Model			Dominant Model		
	Patients with Amnesia/Recall, n	OR	95% CI	*P*	OR	95% CI	*P*
rs73247636 genotype		7.44	2.18–25.39	0.0014	8.39	2.36–29.85	0.0010
A/A	99/56						
A/G	29/3						
G/G	4/0						
rs56278524 genotype		13.19	3.02–57.62	0.0006	15.26	3.42–68.07	0.0004
A/A	94/56						
A/G	33/2						
G/G	5/0						

The recessive tests were not conducted because of insufficient homozygous samples of the minor alleles for the logistic regression analyses. Odds ratios were calculated with the major allele as the reference. OR, odds ratio; CI, confidence interval

## Discussion

In the present study, we comprehensively investigated the association between genetic variants and interindividual differences in the sedative and amnesic effects of midazolam. We found that the *GABRB1* SNPs rs73247636 and rs56278524 were significantly associated with both the sedative and amnesic effects of midazolam in the candidate gene analyses, whereas we did not identify any novel candidate genes using the genome-wide approach. Carriers of the minor G allele of rs73247636 and rs56278524 were more deeply sedated than carriers of homozygous major alleles (Table 2). Moreover, patients with anterograde amnesia were frequently carriers of the minor G allele of these single-nucleotide polymorphisms compared with homozygous major alleles (Table 3). These results suggest that carriers of the minor G allele of rs73247636 and rs56278524 were more sensitive to the effects of midazolam than non-carriers.

The rs73247636 and rs56278524 polymorphisms are located in the third intron region of the *GABRB1* gene, which encodes the GABA_A_ receptor β1 subunit, on chromosome 4 according to the human reference genome GRCh38.p12. According to the Genotype-Tissue Expression Consortium, the rs73247636 SNP significantly affects mRNA expression of the *GABRB1* gene in the human peripheral tibial artery [25, 26]. Unclear are the ways in which these intronic variations lead to changes in the severity of both the sedative and amnesic effects of midazolam. One possibility is that the modification of mRNA expression may affect the physiological and pharmacological properties of the β1 subunit of GABA_A_ receptors. Human genetic association studies have demonstrated that the *GABRB1* gene is associated with bipolar disorder, schizophrenia, alcohol dependence, and alterations of brain function [27–30]. Mutations of *Gabrb1* caused spontaneous GABA ion channel opening in vitro and increased alcohol consumption in mice, which were more sensitive to the sedative and ataxic effects of ethanol [31]. However, the ways in which genetic variations of the *GABRB1* gene affect phenotypic differences remain to be elucidated.

The functions of GABA_A_ receptor subunits have been investigated in studies of mutant mice [12]. The sedative and anterograde amnestic effects of diazepam were mediated by GABA_A_ receptors that contain α1 subunits [32]. Hence, the sedative and amnesic effects of midazolam are presumably mediated by α1-containing GABA_A_ receptors. In humans, a previous study [10] suggested that the rs4263535 SNP of the GABA_A_ receptor α1 subunit gene (*GABRA1*) was associated with deeper sedation by intravenous midazolam. Contrary to our expectation, none of the *GABRA1* polymorphisms, including rs4263535, were associated with the sedative effect of midazolam in the present study (Table S2, Additional file 3). Benzodiazepine binding sites are located between the α and γ subunits, whereas GABA binding sites are located between the α and β subunits [12]. Benzodiazepines allosterically modulate GABA_A_ receptors to increase the frequency of chloride channel opening [33]. One possible interpretation of our results is that interindividual variability in the sedative effect of midazolam is attributable to GABAergic pathways and not to the positive allosteric modulation of GABA_A_ receptors that is mediated by midazolam. Interestingly, sensitivity to the sedative and hypnotic effects of diazepam increased in GABA_A_ α1 subunit knockout mice, whereas the duration of the midazolam-induced loss-of-righting reflex decreased in GABA_A_ β3 subunit knockout mice [13]. No GABA_A_ β1 subunit knockout mice have yet been generated. Thus, our findings refocus attention on the pharmacological role of the β1 subunit.

Previous studies investigated the physiological functions of β1 subunit-containing GABA_A_ receptors. A β1 subunit-specific antagonist reduced the sedative-hypnotic effect of diazepam in mice, but it did not alter the sedative-hypnotic effect of propofol, which activates β1-, β2-, and β3-containing GABA_A_ receptors [34]. These findings are consistent with our candidate gene analysis results. Moreover, β1-containing GABA_A_ receptors modulate the histaminergic sleep pathway in the posterior hypothalamus [35]. An increase in the phosphorylation of β1 subunits was associated with the inhibition of GABAergic currents in vitro, modulated by the orexin-mediated pathway, which regulates sleep and wakefulness [36]. These findings indicate that β1 subunits may be involved in regulating sleep and wakefulness. Furthermore, although the sedative effect of diazepam is mediated by α1-containing GABA_A_ receptors, α1 subunits appear to not be involved in the hypnotic effect of diazepam because the diazepam-induced changes in sleep electroencephalograms were mediated by GABA_A_ receptors that did not contain α1 subunits [37]. Given these results, β1 subunits may be associated with the hypnotic effect of midazolam rather than its sedative effect, although the hypnotic and sedative effects of midazolam are clinically indistinguishable.

The present study has several limitations. First, the sample size was not strictly calculated. This was an exploratory study that performed genome-wide genetic analyses. The sample size was relatively small for genome-wide association studies. Further studies are needed to validate our findings with an appropriate sample size. Second, the results of the presence of amnesia might be affected by the total doses of midazolam and propofol, duration of the dental procedure, and the type of dental procedure. Although unclear was how much propofol was necessary to induce retrograde amnesia, all of the patients were able to recall our instructions that were given before sedation. Additionally, the Ramsay score results should be independent of these factors because Ramsay scores that were used in the analyses were evaluated only before the dental procedures. Third, we did not use the objective measure of BIS values in the analyses because BIS correlated poorly with the depth of sedation with midazolam [38–40]. The level of sedation was assessed by one dentist anesthesiologist. Additionally, genome-wide genotypes of each patient were inevitably blinded. Although possible confounding effects and bias could be expected, we adjusted for potential confounders as covariates.

In conclusion, we found that the rs73247636 and rs56278524 SNPs of the *GABRB1* gene were associated with interindividual differences in the sedative and amnesic effects of midazolam. Patients who carried minor alleles of these polymorphisms may have greater sensitivity to midazolam. Future studies are needed to explore the mechanism that underlies the association between *GABRB1* genetic variants and interindividual variability in midazolam sensitivity.

## Electronic supplementary material

Below is the link to the electronic supplementary material.

Supplementary Material 1

Supplementary Material 2

Supplementary Material 3

Supplementary Material 4

Supplementary Material 5

Supplementary Material 6

Supplementary Material 7

## Data Availability

The datasets generated and/or analysed during the current study are not publicly available due to the content of personal genetic information but are available from the corresponding author on reasonable request.

## Abbreviations

ASA: American Society of Anesthesiologists
CI: Confidence interval
GABA_A_: γ-aminobutyric acid type A
OR: Odds ratio
SNP: single-nucleotide polymorphism
SpO_2_: oxygen saturation measured by pulse oximetry
