# Supplementary material for: γ-Aminobutyric acid type A receptor β1 subunit gene polymorphisms are associated with the sedative and amnesic effects of midazolam
Source: Mol Brain. 2024 Sep 27;17:70. doi: 10.1186/s13041-024-01141-2 (PMC11428381; doi:10.1186/s13041-024-01141-2)
Supplement: Supplementary file 1 — Supplementary Material 1 [file 13041_2024_1141_MOESM1_ESM.pdf]

Table S1. Top variants with P < 0.0001 from the genome-wide association analyses for the Ramsay sedation score

SNP	Chr	Position	A1/A2	MAF	HWE P	β	95% CI	P-value	Q-value	Nearest gene
**Additive model**										
rs12557060	X	28844532	C/T	0.43	0.35	0.52	0.29 to 0.75	1.9×10^{-5}	0.997	*IL1RAPL1*
rs10927025	1	243475916	T/C	0.18	1	−0.61	−0.88 to −0.33	3.0×10^{-5}	0.997	*SDCCAG8*
rs12512414	4	97345253	A/G	0.10	0.44	0.72	0.39 to 1.05	3.7×10^{-5}	0.997	*STPG2*
rs5748445	22	19815256	C/T	0.14	1	0.64	0.35 to 0.94	3.8×10^{-5}	0.997	*GNB1L*
rs5943563	X	28852765	A/G	0.35	0.33	0.51	0.27 to 0.75	4.4×10^{-5}	0.997	*IL1RAPL1*
rs13272355	8	638596	T/C	0.06	0.54	0.91	0.48 to 1.34	4.7×10^{-5}	0.997	*ERICH1*
rs55760674	4	167302958	C/T	0.07	1	−0.86	−1.27 to −0.45	5.4×10^{-5}	0.997	*SPOCK3*
rs9457608	6	159346508	G/A	0.08	0.61	−0.87	−1.28 to −0.46	5.8×10^{-5}	0.997	*LINC02529*
rs6751855	2	191020045	G/A	0.14	0.38	0.67	0.35 to 0.99	6.0×10^{-5}	0.997	*LOC105373805*
rs10272701	7	25871413	C/T	0.30	0.30	0.50	0.26 to 0.74	6.3×10^{-5}	0.997	*LOC105375199*
rs35492283	19	47708982	A/G	0.14	1	−0.64	−0.95 to −0.33	7.0×10^{-5}	0.997	*EHD2*
rs6503113	17	8395675	A/G	0.28	0.21	−0.46	−0.69 to −0.24	7.1×10^{-5}	0.997	*RNF222*
rs116275906	5	178487545	T/C	0.05	1	−1.00	−1.49 to −0.51	8.3×10^{-5}	0.997	*COL23A1*
rs3901366	11	120537362	A/G	0.32	0.51	0.45	0.23 to 0.67	8.4×10^{-5}	0.997	*GRIK4*
rs7255497	19	14271968	C/T	0.14	0.22	0.60	0.31 to 0.89	8.4×10^{-5}	0.997	*LINC01841*
rs56278524	4	47110636	A/G	0.12	0.15	0.63	0.32 to 0.93	9.4×10^{-5}	0.997	*GABRB1*
rs7888988	X	32857960	T/C	0.12	1	0.74	0.38 to 1.11	9.6×10^{-5}	0.997	*DMD*
**Dominant model**										
rs12044321	1	229820178	C/T	0.32	0.06	0.67	0.37 to 0.96	1.5×10^{-5}	0.998	*LOC107985464*
rs12633751	3	2875771	A/G	0.31	0.61	0.67	0.37 to 0.97	1.7×10^{-5}	0.998	*CNTN4*
rs6668794	1	94669347	A/G	0.25	0.85	0.67	0.37 to 0.97	2.3×10^{-5}	0.998	*SLC44A3-AS1*
rs6503113	17	8395675	A/G	0.28	0.21	−0.66	−0.96 to −0.36	2.5×10^{-5}	0.998	*RNF222*
rs12131794	1	219592884	C/A	0.28	1	−0.66	−0.96 to −0.35	3.4×10^{-5}	0.998	*ZC3H11B*
rs13272355	8	638596	T/C	0.06	0.54	0.99	0.53 to 1.44	3.6×10^{-5}	0.998	*ERICH1*
rs6751855	2	191020045	G/A	0.14	0.38	0.73	0.39 to 1.07	3.6×10^{-5}	0.998	*LOC105373805*
rs58973247	3	2862266	T/G	0.30	0.86	0.64	0.34 to 0.94	4.7×10^{-5}	0.998	*CNTN4*
rs5748445	22	19815256	C/T	0.14	1	0.71	0.37 to 1.05	5.2×10^{-5}	0.998	*GNB1L*
rs9457608	6	159346508	G/A	0.08	0.61	−0.87	−1.28 to −0.46	5.8×10^{-5}	0.998	*LINC02529*
rs56216975	4	11265906	A/G	0.34	1	0.62	0.32 to 0.92	8.2×10^{-5}	0.998	*LOC105374488*
rs62333796	4	178947288	G/A	0.10	0.70	0.77	0.39 to 1.14	8.3×10^{-5}	0.998	*LOC105377563*
rs116275906	5	178487545	T/C	0.05	1	−1.00	−1.49 to −0.51	8.3×10^{-5}	0.998	*COL23A1*
rs10927025	1	243475916	T/C	0.18	1	−0.66	−0.98 to −0.33	9.3×10^{-5}	0.998	*SDCCAG8*
rs6461068	7	14094221	A/G	0.20	0.49	−0.64	−0.95 to −0.33	9.4×10^{-5}	0.998	*RPL6P21*
rs35492283	19	47708982	A/G	0.14	1	−0.69	−1.04 to −0.35	9.7×10^{-5}	0.998	*EHD2*
rs55760674	4	167302958	C/T	0.07	1	−0.88	−1.32 to −0.45	9.9×10^{-5}	0.998	*SPOCK3*
**Recessive model**										
rs9323838	14	89253256	C/T	0.39	0.65	−0.95	−1.36 to −0.55	6.8×10^{-6}	0.855	*FOXN3*
rs12512414	4	97345253	A/G	0.10	0.44	2.76	1.58 to 3.94	8.7×10^{-6}	0.855	*STPG2*
rs10414604	19	14258950	A/G	0.14	1	2.37	1.34 to 3.39	1.1×10^{-5}	0.855	*LINC01841*
rs3800861	7	158110786	C/T	0.36	0.43	−0.96	−1.39 to −0.54	1.3×10^{-5}	0.855	*PTPRN2*
rs4670281	2	33766949	G/A	0.35	0.03	1.20	0.67 to 1.74	1.8×10^{-5}	0.855	*LINC01317*
rs7255497	19	14271968	C/T	0.14	0.22	1.89	1.04 to 2.74	2.0×10^{-5}	0.855	*LINC01841*
rs58572023	2	33771705	T/C	0.39	0.09	1.02	0.56 to 1.48	2.2×10^{-5}	0.855	*LINC01317*
rs6585674	10	120898505	C/T	0.32	0.14	1.20	0.65 to 1.75	3.2×10^{-5}	0.920	*WDR11*
rs433201	4	106930118	G/A	0.39	0.54	0.87	0.46 to 1.27	3.8×10^{-5}	0.920	*DKK2*
rs35941482	8	16097502	T/C	0.44	0.31	0.77	0.41 to 1.14	4.5×10^{-5}	0.920	*MSR1*
rs6123098	20	51904751	G/A	0.48	0.88	−0.74	−1.09 to −0.38	6.2×10^{-5}	0.920	*RNU7-6P*
rs17656349	5	150226431	T/C	0.35	0.63	−0.99	−1.47 to −0.52	6.4×10^{-5}	0.920	*CAMK2A*
rs2241695	5	150223261	T/C	0.34	0.87	−0.99	−1.47 to −0.52	6.4×10^{-5}	0.920	*CAMK2A*
rs2253926	14	61620997	T/C	0.37	0.65	−0.92	−1.36 to −0.47	7.2×10^{-5}	0.920	*FLJ22447*
rs10825792	10	56404556	G/A	0.50	0.66	−0.71	−1.06 to −0.37	7.8×10^{-5}	0.920	*ZWINT*
rs11185296	1	108171530	G/A	0.30	0.60	1.15	0.59 to 1.70	8.8×10^{-5}	0.920	*SLC25A24*
rs12419602	11	5856749	T/A	0.34	0.11	0.87	0.45 to 1.31	9.5×10^{-5}	0.920	*OR52E8*

The regression coefficients (β) represent the effect size and direction with the major allele as the reference allele.

SNP, single-nucleotide polymorphism; Chr, chromosome; Position, chromosomal position in Genome Reference Consortium Human Build 38; A1, major allele; A2, minor allele; MAF, minor allele frequency; HWE, Hardy-Weinberg equilibrium exact test; β, regression coefficient; CI, confidence interval; Q, false discovery rate P.
