# Supplementary material for: γ-Aminobutyric acid type A receptor β1 subunit gene polymorphisms are associated with the sedative and amnesic effects of midazolam
Source: Mol Brain. 2024 Sep 27;17:70. doi: 10.1186/s13041-024-01141-2 (PMC11428381; doi:10.1186/s13041-024-01141-2)

## Fig. S1.

Quantile-quantile plots for the genome-wide association analyses of Ramsay sedation scores in (A) the additive model, (B) the dominant model, and (C) the recessive model. Observed *P* values are plotted against expected *P* values.

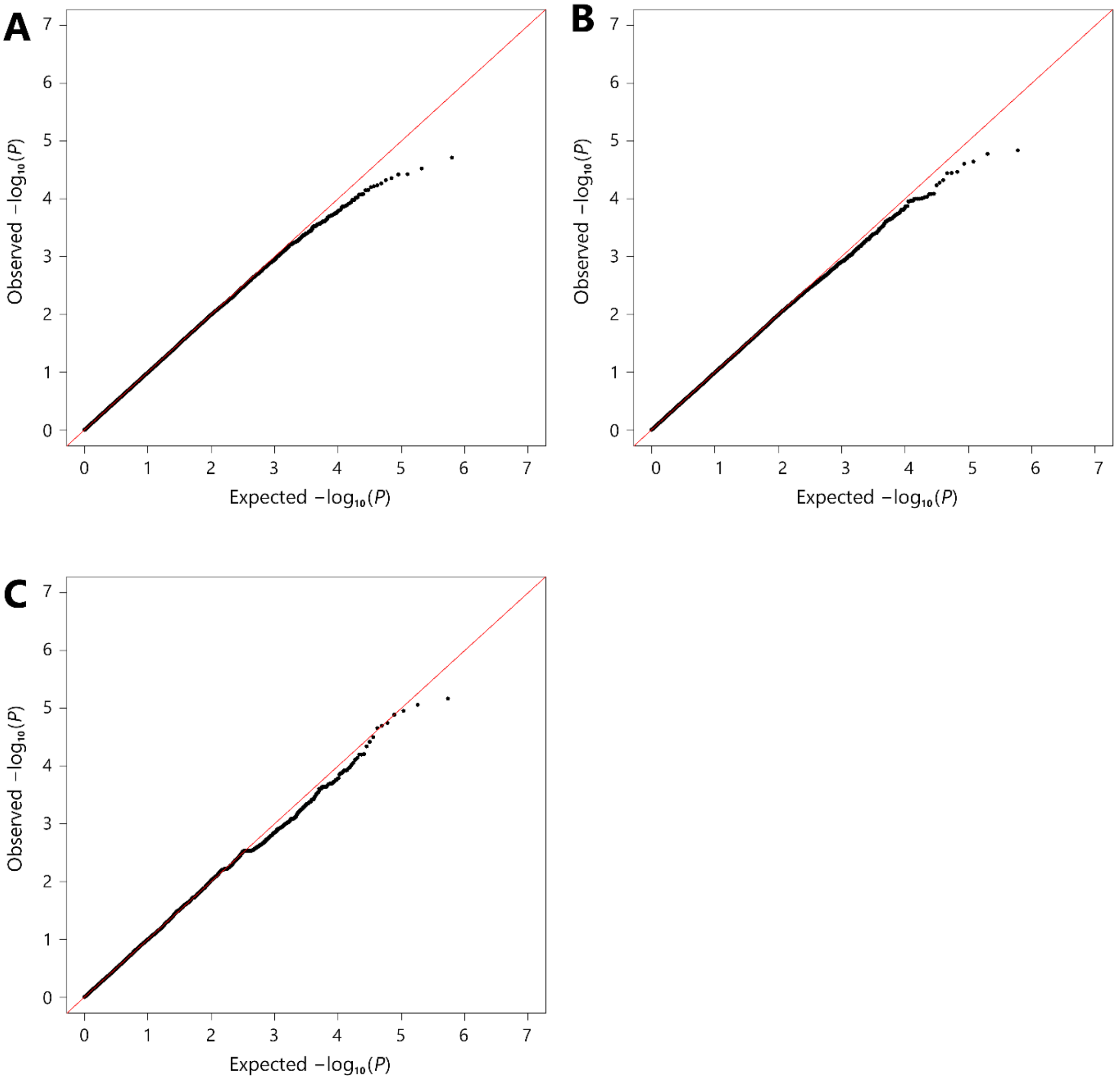

Supplement: Supplementary file 2 — Supplementary Material 2 [file 13041_2024_1141_MOESM2_ESM.pdf]
