# Supplementary material for: γ-Aminobutyric acid type A receptor β1 subunit gene polymorphisms are associated with the sedative and amnesic effects of midazolam
Source: Mol Brain. 2024 Sep 27;17:70. doi: 10.1186/s13041-024-01141-2 (PMC11428381; doi:10.1186/s13041-024-01141-2)
Supplement: Supplementary file 3 — Supplementary Material 3 [file 13041_2024_1141_MOESM3_ESM.pdf]

Table S2. All results from the candidate gene analyses for the Ramsay sedation score

							Additive model				Dominant model				Recessive model			
**Gene**	**Chr**	**SNP**	**Position**	**A1/A2**	**MAF**	**HWE *P***	***β***	**95% CI**	***P***	***Q***	***β***	**95% CI**	***P***	***Q***	***β***	**95% CI**	***P***	***Q***
GABRA1	5	rs4367330	161857776	C/A	0.22	0.83	0.07	−0.20 to 0.34	0.614	0.963	0.14	−0.18 to 0.46	0.381	0.957	−0.28	−1.06 to 0.50	0.486	0.951
		rs4263535	161857823	G/A	0.50	1	−0.05	−0.27 to 0.18	0.687	0.963	−0.06	−0.42 to 0.30	0.740	0.974	−0.06	−0.42 to 0.30	0.746	0.993
		rs10068980	161860841	G/A	0.39	1	0.00	−0.22 to 0.23	0.986	0.996	0.00	−0.33 to 0.32	0.978	0.994	0.02	−0.42 to 0.45	0.945	0.993
		rs12653365	161861618	C/T	0.15	0.77	0.05	−0.26 to 0.37	0.738	0.965	0.09	−0.25 to 0.44	0.597	0.974	−0.37	−1.62 to 0.87	0.558	0.980
		rs7734447	161865032	G/A	0.36	0.21	0.05	−0.19 to 0.29	0.697	0.965	0.07	−0.24 to 0.39	0.648	0.974	0.02	−0.48 to 0.53	0.929	0.993
		rs4254937	161870930	C/A	0.34	0.20	0.07	−0.17 to 0.32	0.563	0.960	0.13	−0.19 to 0.44	0.435	0.957	−0.01	−0.55 to 0.52	0.957	0.993
		rs6894357	161873800	A/G	0.37	0.16	0.08	−0.16 to 0.32	0.525	0.960	0.11	−0.21 to 0.43	0.487	0.957	0.06	−0.44 to 0.56	0.816	0.993
		rs4428455	161875205	G/A	0.10	0.11	0.06	−0.29 to 0.41	0.726	0.965	0.16	−0.25 to 0.56	0.450	0.957	−0.52	−1.60 to 0.57	0.353	0.941
		rs10042696	161879765	C/T	0.35	0.20	0.10	−0.14 to 0.35	0.408	0.960	0.16	−0.15 to 0.48	0.309	0.957	0.03	−0.49 to 0.55	0.921	0.993
		rs12187575	161879940	C/A	0.25	0.70	−0.07	−0.33 to 0.19	0.604	0.963	−0.03	−0.35 to 0.29	0.854	0.974	−0.34	−1.04 to 0.35	0.334	0.941
		rs11135172	161882746	T/G	0.25	0.70	−0.07	−0.33 to 0.19	0.604	0.963	−0.03	−0.35 to 0.29	0.854	0.974	−0.34	−1.04 to 0.35	0.334	0.941
		rs12658807	161882996	A/G	0.14	1	0.02	−0.31 to 0.34	0.920	0.986	0.05	−0.30 to 0.40	0.783	0.974	−0.37	−1.62 to 0.87	0.558	0.980
		rs77662355	161885473	G/A	0.17	1	0.02	−0.28 to 0.31	0.908	0.986	0.05	−0.29 to 0.38	0.779	0.974	−0.22	−1.20 to 0.76	0.661	0.993
		rs4554269	161886153	T/C	0.49	0.56	−0.08	−0.30 to 0.15	0.504	0.960	0.13	−0.23 to 0.49	0.480	0.957	−0.34	−0.71 to 0.02	0.068	0.933
		rs1350372	161887119	A/G	0.37	0.16	0.08	−0.16 to 0.32	0.525	0.960	0.11	−0.21 to 0.43	0.487	0.957	0.06	−0.44 to 0.56	0.816	0.993
		rs12189424	161889590	G/A	0.49	0.56	−0.08	−0.30 to 0.15	0.504	0.960	0.13	−0.23 to 0.49	0.480	0.957	−0.34	−0.71 to 0.02	0.068	0.933
		rs1157122	161892308	T/C	0.25	0.56	−0.03	−0.28 to 0.22	0.805	0.973	−0.04	−0.35 to 0.28	0.804	0.974	−0.04	−0.65 to 0.58	0.902	0.993
		rs6894517	161894462	A/C	0.25	0.56	−0.03	−0.28 to 0.22	0.805	0.973	−0.04	−0.35 to 0.28	0.804	0.974	−0.04	−0.65 to 0.58	0.902	0.993
		rs2279020	161895883	A/G	0.49	0.56	−0.08	−0.30 to 0.15	0.504	0.960	0.13	−0.23 to 0.49	0.480	0.957	−0.34	−0.71 to 0.02	0.068	0.933
		rs2290732	161897892	G/A	0.49	0.77	−0.09	−0.31 to 0.14	0.448	0.960	0.09	−0.26 to 0.45	0.609	0.974	−0.34	−0.70 to 0.03	0.073	0.933
		rs998754	161898928	G/T	0.49	0.56	−0.08	−0.30 to 0.15	0.504	0.960	0.13	−0.23 to 0.49	0.480	0.957	−0.34	−0.71 to 0.02	0.068	0.933
		rs2290733	161899351	T/C	0.17	1	0.02	−0.28 to 0.31	0.908	0.986	0.05	−0.29 to 0.38	0.779	0.974	−0.22	−1.20 to 0.76	0.661	0.993
GABRA2	4	rs534459	46254788	C/T	0.31	0.17	0.10	−0.13 to 0.33	0.386	0.960	0.15	−0.16 to 0.46	0.345	0.957	0.09	−0.40 to 0.59	0.712	0.993
		rs79526700	46257987	C/T	0.07	1	−0.07	−0.53 to 0.39	0.763	0.965	−0.07	−0.53 to 0.39	0.763	0.974	NA	NA	NA	NA
		rs279858	46312576	T/C	0.43	0.24	−0.05	−0.26 to 0.17	0.665	0.963	0.05	−0.28 to 0.38	0.774	0.974	−0.22	−0.60 to 0.16	0.265	0.941
		rs77166883	46314374	T/C	0.18	1	−0.40	−0.68 to −0.12	0.006	0.347	−0.47	−0.80 to −0.15	0.004	0.246	−0.42	−1.31 to 0.47	0.357	0.941
		rs279844	46327638	A/T	0.47	1	−0.09	−0.31 to 0.13	0.437	0.960	0.00	−0.35 to 0.35	0.984	0.994	−0.25	−0.63 to 0.12	0.182	0.941
		rs426463	46343943	A/C	0.47	0.89	−0.08	−0.30 to 0.15	0.500	0.960	0.03	−0.33 to 0.38	0.876	0.974	−0.25	−0.62 to 0.13	0.194	0.941
		rs74607062	46368451	C/T	0.23	0.68	0.09	−0.18 to 0.35	0.533	0.960	0.18	−0.13 to 0.50	0.257	0.944	−0.39	−1.17 to 0.38	0.320	0.941
		rs28728066	46382819	A/G	0.05	1	0.17	−0.32 to 0.67	0.495	0.960	0.17	−0.32 to 0.67	0.495	0.957	NA	NA	NA	NA
GABRA3	X	rs5925128	152193097	G/T	0.16	1	−0.13	−0.47 to 0.22	0.474	0.960								
		rs73619378	152200560	C/T	0.23	0.78	−0.12	−0.41 to 0.17	0.420	0.960								
		rs5925140	152202420	C/T	0.30	0.52	0.20	−0.07 to 0.48	0.149	0.855								
		rs7055052	152204904	C/T	0.22	0.78	0.00	−0.29 to 0.30	0.982	0.996								
		rs12687298	152211186	G/A	0.43	0.34	−0.11	−0.35 to 0.13	0.369	0.960								
		rs994424	152213308	T/C	0.48	0.58	−0.15	−0.39 to 0.09	0.229	0.960								

(Continued)

Table S2. *(Continued)*

Gene	Chr	SNP	Position	A1/A2	MAF	HWE *P*	Additive model *β*	Additive model 95% CI	Additive model *P*	Additive model *Q*	Dominant model *β*	Dominant model 95% CI	Dominant model *P*	Dominant model *Q*	Recessive model *β*	Recessive model 95% CI	Recessive model *P*	Recessive model *Q*
GABRA3	X	rs5970231	152225777	C/T	0.43	0.34	−0.11	−0.35 to 0.13	0.369	0.960								
		rs5970232	152228948	T/C	0.48	0.58	−0.15	−0.39 to 0.09	0.229	0.960								
		rs2194897	152244632	G/C	0.50	0.45	0.11	−0.13 to 0.35	0.387	0.960								
		rs5970242	152245497	G/A	0.48	0.58	−0.14	−0.38 to 0.10	0.245	0.960								
		rs5970244	152246715	C/A	0.49	0.71	0.11	−0.13 to 0.36	0.376	0.960								
		rs12006612	152261256	A/G	0.14	0.67	−0.06	−0.42 to 0.29	0.721	0.965								
		rs1602624	152269948	A/G	0.19	0.51	−0.10	−0.41 to 0.21	0.535	0.960								
		rs11796898	152276279	C/T	0.19	0.49	−0.11	−0.42 to 0.21	0.509	0.960								
		rs13441023	152286778	T/C	0.19	0.74	−0.07	−0.38 to 0.24	0.642	0.963								
		rs5925155	152289446	G/A	0.09	1	0.05	−0.39 to 0.48	0.832	0.978								
		rs875478	152296606	A/C	0.24	0.78	−0.11	−0.39 to 0.18	0.477	0.960								
		rs5969888	152300285	G/A	0.24	0.78	−0.11	−0.39 to 0.18	0.477	0.960								
		rs1492295	152308652	A/C	0.24	0.78	−0.11	−0.39 to 0.18	0.477	0.960								
		rs5970269	152314271	G/A	0.24	0.78	−0.11	−0.39 to 0.18	0.477	0.960								
		rs1009386	152352718	T/G	0.24	0.78	−0.11	−0.39 to 0.18	0.477	0.960								
		rs11795489	152366136	T/C	0.12	0.37	−0.03	−0.39 to 0.33	0.872	0.986								
		rs11796856	152366251	C/T	0.12	0.37	−0.03	−0.39 to 0.33	0.872	0.986								
		rs389292	152372982	C/T	0.18	1	−0.26	−0.58 to 0.06	0.109	0.763								
		rs1229423	152380249	C/T	0.45	1	0.09	−0.16 to 0.33	0.488	0.960								
		rs4828589	152388676	G/A	0.18	1	−0.26	−0.58 to 0.06	0.109	0.763								
		rs5970292	152392396	G/A	0.24	1	0.11	−0.18 to 0.39	0.470	0.960								
		rs6526104	152395450	C/A	0.25	1	0.10	−0.19 to 0.39	0.493	0.960								
		rs5002366	152404735	A/T	0.24	1	0.09	−0.20 to 0.37	0.559	0.960								
		rs4828696	152413524	T/C	0.24	1	0.09	−0.20 to 0.37	0.559	0.960								
		rs6526107	152414344	T/C	0.46	0.85	0.06	−0.19 to 0.30	0.635	0.963								
		rs6627588	152425875	A/C	0.25	1	0.06	−0.23 to 0.34	0.689	0.963								
		rs1109840	152429171	G/A	0.17	1	0.01	−0.32 to 0.33	0.974	0.996								
		rs28868684	152431397	G/A	0.11	0.35	0.05	−0.36 to 0.45	0.817	0.975								
		rs6526110	152434786	G/A	0.50	0.46	0.01	−0.23 to 0.25	0.918	0.986								
		rs7065528	152436694	G/A	0.50	0.46	0.01	−0.23 to 0.25	0.918	0.986								
		rs1112122	152437532	T/G	0.32	0.67	0.15	−0.10 to 0.41	0.241	0.960								
		rs6627594	152448236	G/A	0.37	1	0.12	−0.14 to 0.37	0.374	0.960								
		rs6627595	152449531	C/G	0.37	1	0.09	−0.16 to 0.34	0.486	0.960								
GABRA5	15	rs2075716	26869757	C/T	0.17	0.21	0.11	−0.19 to 0.42	0.465	0.960	0.10	−0.23 to 0.43	0.559	0.974	0.49	−0.75 to 1.74	0.438	0.945
		rs8039341	26869904	C/T	0.49	0.11	−0.01	−0.24 to 0.22	0.929	0.986	−0.01	−0.38 to 0.37	0.975	0.994	−0.02	−0.40 to 0.36	0.910	0.993
		rs78589962	26870293	G/A	0.12	0.73	−0.21	−0.55 to 0.14	0.241	0.960	−0.22	−0.60 to 0.16	0.268	0.957	−0.41	−1.65 to 0.84	0.525	0.966

(Continued)

Table S2. *(Continued)*

							Additive model				Dominant model				Recessive model			
**Gene**	**Chr**	**SNP**	**Position**	**A1/A2**	**MAF**	**HWE *P***	***β***	**95% CI**	***P***	***Q***	***β***	**95% CI**	***P***	***Q***	***β***	**95% CI**	***P***	***Q***
GABRA5	15	rs28396829	26870418	G/T	0.37	0.76	−0.18	−0.40 to 0.05	0.121	0.774	−0.35	−0.66 to −0.04	0.030	0.485	−0.01	−0.45 to 0.44	0.979	0.993
		rs78575803	26870462	G/A	0.13	0.09	−0.07	−0.38 to 0.24	0.651	0.963	−0.20	−0.57 to 0.18	0.307	0.957	0.52	−0.37 to 1.42	0.253	0.941
		rs7173687	26871571	A/G	0.35	0.64	−0.13	−0.36 to 0.10	0.258	0.960	−0.16	−0.48 to 0.15	0.309	0.957	−0.19	−0.65 to 0.27	0.418	0.945
		rs28678281	26875517	G/A	0.25	0.85	−0.08	−0.32 to 0.17	0.554	0.960	0.04	−0.27 to 0.35	0.811	0.974	−0.61	−1.22 to 0.00	0.052	0.933
		rs28588258	26876855	C/T	0.15	0.05	−0.05	−0.33 to 0.24	0.754	0.965	−0.11	−0.46 to 0.25	0.553	0.974	0.18	−0.60 to 0.95	0.652	0.993
		rs9744196	26888322	C/A	0.34	0.52	0.21	−0.03 to 0.44	0.093	0.749	0.14	−0.17 to 0.46	0.377	0.957	0.57	0.06 to 1.08	0.030	0.933
		rs12909683	26894550	A/G	0.35	0.15	−0.12	−0.34 to 0.09	0.270	0.960	−0.12	−0.43 to 0.19	0.441	0.957	−0.25	−0.69 to 0.18	0.259	0.941
		rs150484972	26895982	T/C	0.16	0.42	0.15	−0.16 to 0.46	0.338	0.960	0.13	−0.21 to 0.46	0.465	0.957	0.73	−0.51 to 1.97	0.251	0.941
		rs9745027	26897119	T/G	0.42	0.77	−0.19	−0.41 to 0.04	0.104	0.759	−0.20	−0.53 to 0.12	0.224	0.913	−0.31	−0.72 to 0.10	0.145	0.933
		rs148892885	26904588	A/G	0.09	0.37	−0.09	−0.49 to 0.32	0.672	0.963	−0.09	−0.49 to 0.32	0.672	0.974	NA	NA	NA	NA
		rs6606901	26907849	G/A	0.42	0.66	−0.19	−0.42 to 0.03	0.095	0.749	−0.22	−0.55 to 0.12	0.205	0.913	−0.31	−0.72 to 0.10	0.142	0.933
		rs143338755	26909482	A/G	0.07	1	0.04	−0.38 to 0.47	0.843	0.978	0.05	−0.40 to 0.51	0.820	0.974	−0.09	−2.26 to 2.07	0.932	0.993
		rs4073083	26909926	G/A	0.11	1	0.04	−0.31 to 0.39	0.836	0.978	0.04	−0.34 to 0.42	0.834	0.974	0.04	−1.48 to 1.57	0.956	0.993
		rs35462260	26911079	G/A	0.27	0.15	0.08	−0.19 to 0.34	0.575	0.960	0.02	−0.29 to 0.34	0.882	0.974	0.42	−0.29 to 1.12	0.247	0.941
		rs61999575	26911685	T/C	0.31	0.40	−0.02	−0.27 to 0.24	0.901	0.986	−0.03	−0.35 to 0.28	0.843	0.974	0.02	−0.56 to 0.60	0.939	0.993
		rs7172131	26911893	G/A	0.44	0.77	0.07	−0.16 to 0.29	0.569	0.960	0.03	−0.31 to 0.37	0.857	0.974	0.16	−0.24 to 0.56	0.423	0.945
		rs78787728	26912169	A/G	0.17	0.80	−0.10	−0.39 to 0.19	0.496	0.960	−0.05	−0.40 to 0.29	0.756	0.974	−0.57	−1.46 to 0.31	0.207	0.941
		rs7172750	26912193	G/A	0.46	1	0.11	−0.11 to 0.34	0.317	0.960	0.22	−0.13 to 0.56	0.215	0.913	0.07	−0.32 to 0.45	0.725	0.993
		rs6606854	26914049	T/C	0.45	1	−0.13	−0.35 to 0.09	0.248	0.960	−0.16	−0.50 to 0.17	0.346	0.957	−0.19	−0.58 to 0.20	0.347	0.941
		rs35399885	26915388	T/C	0.45	1	−0.14	−0.36 to 0.08	0.227	0.960	−0.21	−0.55 to 0.13	0.221	0.913	−0.14	−0.53 to 0.24	0.473	0.945
		rs76674430	26916914	G/A	0.16	0.42	−0.06	−0.36 to 0.23	0.683	0.963	−0.04	−0.38 to 0.31	0.834	0.974	−0.32	−1.21 to 0.58	0.488	0.951
		rs35666182	26919421	C/T	0.15	0.78	−0.10	−0.40 to 0.20	0.509	0.960	−0.04	−0.39 to 0.31	0.825	0.974	−0.75	−1.72 to 0.22	0.132	0.933
		rs61999585	26920032	T/C	0.15	0.78	−0.08	−0.38 to 0.22	0.583	0.960	−0.11	−0.45 to 0.24	0.539	0.974	−0.02	−1.00 to 0.96	0.966	0.993
		rs7163259	26925838	C/T	0.07	0.57	−0.33	−0.77 to 0.11	0.149	0.855	−0.28	−0.75 to 0.18	0.234	0.913	−1.74	−3.90 to 0.41	0.115	0.933
		rs7163639	26926001	C/T	0.07	0.57	−0.33	−0.77 to 0.11	0.149	0.855	−0.28	−0.75 to 0.18	0.234	0.913	−1.74	−3.90 to 0.41	0.115	0.933
		rs8031094	26928729	G/A	0.25	1.0	−0.20	−0.45 to 0.05	0.123	0.774	−0.13	−0.44 to 0.19	0.427	0.957	−0.74	−1.37 to −0.11	0.023	0.933
		rs8028947	26928735	T/C	0.09	0.17	−0.35	−0.71 to 0.02	0.065	0.749	−0.26	−0.68 to 0.16	0.229	0.913	−1.71	−2.94 to −0.49	0.007	0.933
		rs61999604	26929463	T/G	0.14	0.55	−0.23	−0.54 to 0.08	0.141	0.855	−0.27	−0.62 to 0.09	0.140	0.841	−0.31	−1.30 to 0.67	0.535	0.966
		rs12443167	26931290	C/T	0.17	0.61	−0.15	−0.45 to 0.15	0.334	0.960	−0.19	−0.52 to 0.14	0.269	0.957	0.07	−1.03 to 1.16	0.906	0.993
		rs12904832	26934288	G/A	0.34	0.52	−0.08	−0.32 to 0.16	0.504	0.960	−0.08	−0.39 to 0.23	0.616	0.974	−0.16	−0.68 to 0.36	0.537	0.966
		rs12911026	26935653	A/G	0.34	0.87	0.22	−0.01 to 0.45	0.065	0.749	0.30	−0.01 to 0.61	0.063	0.550	0.24	−0.25 to 0.74	0.339	0.941
		rs11263708	26935768	C/T	0.45	0.88	−0.19	−0.41 to 0.03	0.099	0.749	−0.20	−0.53 to 0.14	0.256	0.944	−0.32	−0.70 to 0.07	0.111	0.933
		rs34037073	26936358	A/C	0.25	0.56	−0.15	−0.41 to 0.11	0.256	0.960	−0.21	−0.52 to 0.10	0.186	0.879	−0.03	−0.73 to 0.67	0.930	0.993
		rs1864793	26938442	C/T	0.20	0.66	0.17	−0.10 to 0.43	0.226	0.960	0.19	−0.13 to 0.51	0.252	0.944	0.27	−0.46 to 1.00	0.472	0.945
		rs4887530	26945074	T/C	0.17	0.21	−0.03	−0.34 to 0.28	0.852	0.979	0.00	−0.34 to 0.33	0.977	0.994	−0.42	−1.68 to 0.83	0.511	0.961

(Continued)

Table S2. *(Continued)*

Gene	Chr	SNP	Position	A1/A2	MAF	HWE	Additive model				Dominant model				Recessive model			
						P	*β*	95% CI	*P*	*Q*	*β*	95% CI	*P*	*Q*	*β*	95% CI	*P*	*Q*
GABRB1	4	rs62303735	46997472	G/A	0.16	1.0	0.00	−0.31 to 0.30	0.995	0.997	0.00	−0.34 to 0.34	0.991	0.997	0.01	−1.07 to 1.09	0.987	0.994
		rs10938467	47000034	A/G	0.10	0.02	0.14	−0.20 to 0.48	0.417	0.960	0.11	−0.30 to 0.52	0.598	0.974	0.54	−0.43 to 1.51	0.274	0.941
		rs13116518	47001116	T/C	0.31	0.61	0.10	−0.13 to 0.34	0.390	0.960	0.11	−0.20 to 0.42	0.498	0.957	0.20	−0.31 to 0.71	0.446	0.945
		rs1512142	47002288	C/T	0.40	0.65	0.15	−0.08 to 0.38	0.191	0.960	0.10	−0.23 to 0.42	0.564	0.974	0.38	−0.05 to 0.82	0.086	0.933
		rs13139021	47004382	C/T	0.38	0.76	−0.05	−0.28 to 0.18	0.681	0.963	−0.17	−0.49 to 0.15	0.305	0.957	0.15	−0.31 to 0.60	0.526	0.966
		rs10015366	47055742	G/A	0.32	0.74	0.35	0.12 to 0.58	0.003	0.320	0.48	0.17 to 0.78	0.003	0.201	0.37	−0.14 to 0.88	0.157	0.933
		rs78665078	47058808	C/T	0.10	0.69	−0.04	−0.41 to 0.34	0.844	0.978	−0.03	−0.44 to 0.38	0.886	0.974	−0.21	−1.74 to 1.32	0.788	0.993
		rs4487315	47068858	T/C	0.18	0.13	−0.19	−0.46 to 0.08	0.172	0.913	−0.23	−0.57 to 0.10	0.179	0.856	−0.27	−1.01 to 0.46	0.463	0.945
		rs117303901	47073405	G/A	0.08	1	−0.08	−0.49 to 0.34	0.716	0.965	−0.06	−0.50 to 0.38	0.797	0.974	−0.69	−2.84 to 1.46	0.532	0.966
		rs4613538	47081703	C/T	0.32	0.87	−0.08	−0.32 to 0.16	0.517	0.960	−0.26	−0.57 to 0.05	0.100	0.715	0.35	−0.17 to 0.87	0.186	0.941
		rs4075285	47084774	A/G	0.08	0.08	0.13	−0.26 to 0.51	0.517	0.960	0.14	−0.31 to 0.60	0.531	0.974	0.23	−1.02 to 1.47	0.723	0.993
		rs4396968	47087167	C/T	0.13	1	0.06	−0.27 to 0.39	0.717	0.965	0.07	−0.29 to 0.42	0.716	0.974	0.08	−1.17 to 1.33	0.904	0.993
		rs73247636	47092489	A/G	0.10	0.13	0.64	0.31 to 0.97	2.1×10^{-4}	**0.039**	0.72	0.34 to 1.10	3.0×10^{-4}	**0.047**	1.03	−0.05 to 2.11	0.064	0.933
		rs1866990	47099206	G/A	0.32	0.41	0.29	0.07 to 0.52	0.012	0.391	0.39	0.08 to 0.70	0.014	0.403	0.37	−0.12 to 0.85	0.142	0.933
		rs10517178	47100976	C/T	0.06	1	−0.08	−0.55 to 0.40	0.746	0.965	−0.08	−0.55 to 0.40	0.746	0.974	NA	NA	NA	NA
		rs971354	47101098	A/G	0.43	0.66	−0.21	−0.43 to 0.02	0.073	0.749	−0.33	−0.66 to 0.00	0.050	0.497	−0.18	−0.59 to 0.24	0.405	0.945
		rs17538945	47108026	A/C	0.09	0.19	0.12	−0.25 to 0.48	0.527	0.960	0.13	−0.29 to 0.54	0.546	0.974	0.23	−1.02 to 1.47	0.723	0.993
		rs56278524	47110636	A/G	0.12	0.15	0.63	0.32 to 0.93	9.4×10^{-5}	**0.035**	0.73	0.37 to 1.10	1.1×10^{-4}	**0.035**	0.94	−0.02 to 1.91	0.058	0.933
		rs1561778	47113922	T/C	0.37	0.28	−0.15	−0.39 to 0.09	0.216	0.960	−0.23	−0.55 to 0.09	0.155	0.841	−0.09	−0.57 to 0.39	0.706	0.993
		rs35262963	47121967	C/T	0.23	0.54	0.34	0.08 to 0.61	0.012	0.391	0.37	0.06 to 0.68	0.022	0.482	0.61	−0.15 to 1.38	0.119	0.933
		rs11941080	47146286	C/T	0.12	0.49	−0.12	−0.45 to 0.22	0.499	0.960	−0.17	−0.55 to 0.22	0.397	0.957	0.11	−0.98 to 1.20	0.840	0.993
		rs11940530	47165298	C/T	0.32	0.10	−0.16	−0.41 to 0.09	0.218	0.960	−0.13	−0.44 to 0.18	0.420	0.957	−0.40	−0.97 to 0.18	0.175	0.941
		rs2119779	47173683	A/G	0.46	0.88	−0.06	−0.28 to 0.16	0.589	0.960	−0.14	−0.48 to 0.20	0.418	0.957	−0.01	−0.39 to 0.38	0.979	0.993
		rs13131816	47181494	C/T	0.46	0.88	−0.06	−0.28 to 0.16	0.589	0.960	−0.14	−0.48 to 0.20	0.418	0.957	−0.01	−0.39 to 0.38	0.979	0.993
		rs79818154	47210610	T/C	0.10	0.10	0.06	−0.29 to 0.40	0.742	0.965	0.03	−0.38 to 0.43	0.893	0.974	0.37	−0.71 to 1.46	0.501	0.951
		rs1372498	47224187	G/A	0.31	0.73	0.06	−0.17 to 0.30	0.596	0.960	0.11	−0.20 to 0.42	0.501	0.957	0.01	−0.51 to 0.53	0.963	0.993
		rs16860087	47227230	C/A	0.31	0.73	0.06	−0.17 to 0.30	0.596	0.960	0.11	−0.20 to 0.42	0.501	0.957	0.01	−0.51 to 0.53	0.963	0.993
		rs78317477	47245568	G/A	0.09	0.15	0.13	−0.25 to 0.50	0.509	0.960	0.07	−0.36 to 0.50	0.736	0.974	0.78	−0.46 to 2.02	0.221	0.941
		rs10026884	47270375	G/A	0.21	0.82	0.01	−0.26 to 0.29	0.927	0.986	−0.01	−0.33 to 0.31	0.946	0.994	0.19	−0.64 to 1.02	0.651	0.993
		rs4396966	47289118	C/T	0.32	0.03	−0.19	−0.41 to 0.03	0.090	0.749	−0.29	−0.60 to 0.02	0.066	0.565	−0.19	−0.64 to 0.26	0.409	0.945
		rs6284	47320202	C/A	0.13	0.52	0.29	−0.03 to 0.61	0.073	0.749	0.38	0.02 to 0.74	0.039	0.485	−0.03	−1.11 to 1.06	0.958	0.993
		rs75249114	47326934	G/A	0.21	0.39	0.22	−0.04 to 0.49	0.098	0.749	0.17	−0.15 to 0.49	0.308	0.957	0.79	0.09 to 1.48	0.028	0.933
		rs7665508	47345272	A/G	0.30	0.06	−0.24	−0.46 to −0.02	0.035	0.659	−0.33	−0.64 to −0.02	0.037	0.485	−0.31	−0.78 to 0.17	0.206	0.941
		rs80034809	47347366	A/C	0.28	0.15	−0.23	−0.46 to 0.00	0.055	0.728	−0.30	−0.61 to 0.01	0.057	0.512	−0.30	−0.81 to 0.22	0.261	0.941
		rs7698812	47366208	A/G	0.16	1	0.02	−0.28 to 0.32	0.885	0.986	0.02	−0.32 to 0.36	0.908	0.974	0.07	−0.90 to 1.05	0.885	0.993
		rs4694845	47366610	C/A	0.16	1	0.02	−0.28 to 0.32	0.889	0.986	0.02	−0.32 to 0.36	0.913	0.974	0.07	−0.90 to 1.05	0.886	0.993

(Continued)

Table S2. *(Continued)*

Gene	Chr	SNP	Position	A1/A2	MAF	HWE	Additive model				Dominant model				Recessive model			
						P	*β*	95% CI	*P*	*Q*	*β*	95% CI	*P*	*Q*	*β*	95% CI	*P*	*Q*
GABRB1	4	rs4695220	47366663	T/C	0.16	1	0.02	−0.28 to 0.32	0.889	0.986	0.02	−0.32 to 0.36	0.913	0.974	0.07	−0.90 to 1.05	0.886	0.993
		rs4694846	47366781	T/C	0.15	1	−0.08	−0.38 to 0.23	0.624	0.963	−0.08	−0.43 to 0.26	0.646	0.974	−0.15	−1.24 to 0.93	0.781	0.993
		rs10938482	47367740	A/C	0.16	1	0.01	−0.29 to 0.32	0.942	0.986	0.02	−0.32 to 0.36	0.913	0.974	−0.05	−1.14 to 1.04	0.929	0.993
		rs7677890	47369572	T/C	0.16	1	0.01	−0.29 to 0.32	0.942	0.986	0.02	−0.32 to 0.36	0.913	0.974	−0.05	−1.14 to 1.04	0.929	0.993
		rs12642873	47370122	C/A	0.30	0.87	0.21	−0.03 to 0.44	0.089	0.749	0.22	−0.09 to 0.53	0.172	0.844	0.40	−0.13 to 0.92	0.141	0.933
		rs58150060	47371383	T/C	0.16	1	0.01	−0.29 to 0.32	0.942	0.986	0.02	−0.32 to 0.36	0.913	0.974	−0.05	−1.14 to 1.04	0.929	0.993
		rs75235555	47381743	T/C	0.16	1	−0.06	−0.37 to 0.24	0.687	0.963	−0.06	−0.40 to 0.28	0.729	0.974	−0.18	−1.26 to 0.90	0.744	0.993
		rs4328889	47386127	G/A	0.17	0.61	−0.04	−0.34 to 0.26	0.781	0.965	−0.08	−0.41 to 0.25	0.630	0.974	0.31	−0.77 to 1.40	0.572	0.993
		rs4407474	47398314	C/A	0.11	1	−0.20	−0.55 to 0.15	0.275	0.960	−0.13	−0.51 to 0.25	0.509	0.957	−1.61	−3.11 to −0.11	0.037	0.933
		rs6817416	47399170	G/A	0.11	1	−0.18	−0.54 to 0.17	0.305	0.960	−0.12	−0.50 to 0.27	0.552	0.974	−1.60	−3.11 to −0.09	0.039	0.933
		rs35082184	47399698	A/G	0.23	0.42	0.16	−0.10 to 0.41	0.223	0.960	0.20	−0.11 to 0.52	0.212	0.913	0.18	−0.47 to 0.83	0.586	0.993
		rs17600651	47408486	G/A	0.12	0.72	−0.07	−0.41 to 0.27	0.691	0.963	0.07	−0.31 to 0.45	0.709	0.974	−1.72	−2.94 to −0.49	0.007	0.933
		rs7659411	47411104	C/T	0.15	0.15	0.30	0.01 to 0.59	0.043	0.659	0.37	0.02 to 0.72	0.040	0.485	0.40	−0.43 to 1.23	0.348	0.941
		rs13106855	47411505	G/A	0.15	0.15	0.30	0.01 to 0.59	0.043	0.659	0.37	0.02 to 0.72	0.040	0.485	0.40	−0.43 to 1.23	0.348	0.941
		rs17653595	47413396	A/G	0.08	0.03	−0.02	−0.39 to 0.34	0.905	0.986	0.11	−0.33 to 0.55	0.622	0.974	−0.87	−1.94 to 0.21	0.116	0.933
		rs75940832	47414119	C/T	0.08	0.03	−0.02	−0.39 to 0.34	0.905	0.986	0.11	−0.33 to 0.55	0.622	0.974	−0.87	−1.94 to 0.21	0.116	0.933
		rs10155404	47419308	G/A	0.31	0.74	0.14	−0.09 to 0.37	0.236	0.960	0.23	−0.08 to 0.54	0.145	0.841	0.05	−0.46 to 0.56	0.844	0.993
		rs10155287	47419413	A/G	0.27	0.36	0.28	0.04 to 0.51	0.023	0.545	0.35	0.04 to 0.66	0.026	0.482	0.36	−0.20 to 0.92	0.205	0.941
		rs10155173	47419634	C/A	0.26	0.35	0.27	0.03 to 0.50	0.029	0.603	0.34	0.03 to 0.65	0.033	0.485	0.36	−0.20 to 0.92	0.205	0.941
		rs4493516	47419941	C/T	0.26	0.46	0.31	0.07 to 0.55	0.013	0.391	0.36	0.05 to 0.67	0.024	0.482	0.51	−0.06 to 1.09	0.083	0.933
		rs13102764	47428910	G/A	0.24	0.43	0.16	−0.09 to 0.41	0.210	0.960	0.22	−0.09 to 0.54	0.168	0.844	0.12	−0.49 to 0.74	0.695	0.993
		rs10470771	47434554	A/G	0.15	0.17	0.28	0.00 to 0.57	0.056	0.728	0.34	−0.01 to 0.69	0.056	0.512	0.40	−0.43 to 1.23	0.348	0.941
GABRB2	5	rs74460025	161295354	A/G	0.11	0.70	−0.08	−0.46 to 0.29	0.654	0.963	−0.01	−0.40 to 0.38	0.959	0.994	−2.56	−4.68 to −0.43	0.020	0.933
		rs1849173	161304157	T/C	0.41	0.46	−0.09	−0.32 to 0.15	0.468	0.960	−0.03	−0.36 to 0.30	0.840	0.974	−0.25	−0.68 to 0.19	0.273	0.941
		rs3850733	161311310	C/A	0.08	1	0.23	−0.17 to 0.64	0.264	0.960	0.21	−0.22 to 0.64	0.335	0.957	1.19	−0.98 to 3.36	0.283	0.941
		rs1816071	161332949	A/G	0.25	0.34	0.15	−0.11 to 0.41	0.266	0.960	0.25	−0.06 to 0.57	0.110	0.752	−0.25	−0.98 to 0.48	0.503	0.951
		rs73316976	161333612	A/G	0.34	0.33	−0.15	−0.37 to 0.08	0.213	0.960	−0.15	−0.46 to 0.17	0.365	0.957	−0.29	−0.75 to 0.18	0.230	0.941
		rs4921190	161333788	C/T	0.08	1	0.30	−0.11 to 0.70	0.154	0.867	0.28	−0.15 to 0.71	0.199	0.910	1.19	−0.98 to 3.36	0.283	0.941
		rs967771	161335167	C/T	0.10	0.11	0.43	0.09 to 0.77	0.015	0.415	0.44	0.04 to 0.84	0.031	0.485	1.03	−0.05 to 2.11	0.064	0.933
		rs9313880	161335530	T/C	0.06	0.54	−0.11	−0.56 to 0.35	0.637	0.963	−0.17	−0.65 to 0.32	0.505	0.957	0.79	−1.37 to 2.95	0.473	0.945
		rs10052441	161336780	A/G	0.06	0.54	−0.11	−0.56 to 0.35	0.637	0.963	−0.17	−0.65 to 0.32	0.505	0.957	0.79	−1.37 to 2.95	0.473	0.945
		rs17521304	161404861	T/C	0.10	0.10	0.46	0.11 to 0.80	0.010	0.391	0.48	0.08 to 0.89	0.020	0.482	1.03	−0.05 to 2.11	0.064	0.933
		rs17059452	161430625	A/G	0.19	1	0.05	−0.24 to 0.33	0.739	0.965	0.04	−0.29 to 0.37	0.814	0.974	0.16	−0.67 to 0.99	0.701	0.993
		rs4403218	161462228	G/A	0.16	0.18	0.10	−0.23 to 0.42	0.550	0.960	0.07	−0.27 to 0.42	0.677	0.974	0.75	−0.78 to 2.28	0.339	0.941
		rs7724146	161467625	T/C	0.24	1	−0.11	−0.37 to 0.15	0.409	0.960	−0.20	−0.51 to 0.11	0.215	0.913	0.17	−0.50 to 0.84	0.621	0.993
		rs2964773	161483206	A/G	0.12	1	−0.04	−0.39 to 0.31	0.808	0.973	−0.05	−0.42 to 0.33	0.802	0.974	−0.03	−1.57 to 1.51	0.968	0.993

(Continued)

Table S2. *(Continued)*

							Additive model				Dominant model				Recessive model			
**Gene**	**Chr**	**SNP**	**Position**	**A1/A2**	**MAF**	**HWE *P***	***β***	**95% CI**	***P***	***Q***	***β***	**95% CI**	***P***	***Q***	***β***	**95% CI**	***P***	***Q***
GABRB2	5	rs6881515	161484962	T/C	0.14	0.14	−0.30	−0.63 to 0.03	0.079	0.749	−0.34	−0.68 to 0.00	0.050	0.497	1.01	−1.16 to 3.18	0.363	0.941
		rs12153038	161494469	C/T	0.14	0.14	−0.30	−0.63 to 0.03	0.079	0.749	−0.34	−0.68 to 0.00	0.050	0.497	1.01	−1.16 to 3.18	0.363	0.941
GABRB3	15	rs3751582	26560917	C/T	0.20	0.51	0.15	−0.14 to 0.43	0.312	0.960	0.13	−0.19 to 0.45	0.424	0.957	0.45	−0.44 to 1.35	0.322	0.941
		rs8030011	26573215	G/A	0.06	1	0.43	−0.05 to 0.91	0.081	0.749	0.43	−0.05 to 0.91	0.081	0.621	NA	NA	NA	NA
		rs74666350	26579330	C/A	0.18	0.81	0.05	−0.25 to 0.34	0.762	0.965	0.07	−0.26 to 0.41	0.679	0.974	−0.10	−1.08 to 0.87	0.835	0.993
		rs17117177	26620485	C/A	0.06	1	0.38	−0.09 to 0.86	0.114	0.767	0.38	−0.09 to 0.86	0.114	0.752	NA	NA	NA	NA
		rs17646626	26622139	G/A	0.13	0.54	0.17	−0.17 to 0.50	0.332	0.960	0.12	−0.23 to 0.48	0.502	0.957	1.19	−0.32 to 2.71	0.124	0.933
		rs12438141	26625455	C/T	0.06	1	0.38	−0.09 to 0.86	0.114	0.767	0.38	−0.09 to 0.86	0.114	0.752	NA	NA	NA	NA
		rs73368376	26641680	A/G	0.09	1	0.38	−0.01 to 0.76	0.057	0.728	0.37	−0.04 to 0.77	0.077	0.612	1.30	−0.84 to 3.44	0.236	0.941
		rs11629819	26649934	C/A	0.08	0.61	−0.06	−0.50 to 0.37	0.773	0.965	−0.06	−0.50 to 0.37	0.773	0.974	NA	NA	NA	NA
		rs12439236	26659541	C/T	0.14	0.36	−0.12	−0.43 to 0.18	0.430	0.960	−0.07	−0.43 to 0.29	0.699	0.974	−0.72	−1.70 to 0.25	0.148	0.933
		rs17738087	26659874	C/T	0.13	0.19	−0.04	−0.36 to 0.28	0.803	0.973	0.05	−0.32 to 0.42	0.800	0.974	−0.72	−1.70 to 0.25	0.148	0.933
		rs2217068	26662532	A/G	0.07	0.60	−0.16	−0.60 to 0.27	0.467	0.960	−0.25	−0.71 to 0.21	0.293	0.957	1.43	−0.72 to 3.57	0.195	0.941
		rs149783889	26667085	C/T	0.05	0.44	0.41	−0.06 to 0.89	0.091	0.749	0.39	−0.13 to 0.90	0.142	0.841	1.59	−0.56 to 3.73	0.149	0.933
		rs2315905	26668545	C/T	0.30	0.87	0.04	−0.20 to 0.28	0.717	0.965	0.04	−0.27 to 0.35	0.794	0.974	0.10	−0.43 to 0.63	0.719	0.993
		rs1346150	26673606	T/C	0.32	0.07	−0.02	−0.24 to 0.20	0.879	0.986	0.02	−0.29 to 0.33	0.907	0.974	−0.11	−0.57 to 0.34	0.626	0.993
		rs4906892	26678501	A/G	0.32	0.10	−0.03	−0.25 to 0.19	0.772	0.965	0.02	−0.29 to 0.33	0.907	0.974	−0.19	−0.65 to 0.28	0.433	0.945
		rs7494844	26678933	T/C	0.06	1	−0.18	−0.67 to 0.30	0.458	0.960	−0.18	−0.67 to 0.30	0.458	0.957	NA	NA	NA	NA
		rs12439751	26680762	G/A	0.08	0.32	0.38	−0.02 to 0.77	0.065	0.749	0.39	−0.05 to 0.83	0.086	0.633	0.85	−0.67 to 2.38	0.275	0.941
		rs10873637	26681618	A/C	0.50	0.89	0.12	−0.10 to 0.34	0.292	0.960	0.29	−0.07 to 0.64	0.119	0.768	0.03	−0.33 to 0.39	0.875	0.993
		rs28536117	26686984	A/C	0.40	1	−0.02	−0.25 to 0.20	0.839	0.978	0.10	−0.23 to 0.42	0.562	0.974	−0.24	−0.66 to 0.17	0.254	0.941
		rs752414	26689854	G/A	0.09	0.65	0.08	−0.30 to 0.46	0.677	0.963	0.07	−0.35 to 0.48	0.751	0.974	0.40	−1.13 to 1.93	0.607	0.993
		rs17738278	26695966	G/A	0.43	0.88	−0.02	−0.24 to 0.21	0.893	0.986	0.04	−0.29 to 0.37	0.830	0.974	−0.11	−0.51 to 0.30	0.612	0.993
		rs981777	26711850	T/C	0.26	0.85	−0.09	−0.34 to 0.16	0.479	0.960	−0.11	−0.42 to 0.20	0.492	0.957	−0.13	−0.77 to 0.52	0.697	0.993
		rs11632969	26713630	G/A	0.41	0.55	−0.10	−0.32 to 0.12	0.380	0.960	−0.09	−0.42 to 0.23	0.576	0.974	−0.19	−0.60 to 0.22	0.357	0.941
		rs74829653	26722299	T/C	0.32	0.62	−0.03	−0.27 to 0.21	0.820	0.975	0.02	−0.30 to 0.33	0.907	0.974	−0.19	−0.72 to 0.34	0.486	0.951
		rs36053947	26722366	C/T	0.37	0.88	−0.02	−0.26 to 0.21	0.836	0.978	−0.05	−0.37 to 0.27	0.761	0.974	0.01	−0.46 to 0.47	0.979	0.993
		rs12904097	26722624	C/T	0.37	0.88	−0.02	−0.26 to 0.21	0.836	0.978	−0.05	−0.37 to 0.27	0.761	0.974	0.01	−0.46 to 0.47	0.979	0.993
		rs8043440	26727939	T/C	0.32	0.87	−0.08	−0.32 to 0.16	0.536	0.960	−0.06	−0.37 to 0.26	0.716	0.974	−0.20	−0.72 to 0.33	0.462	0.945
		rs6576602	26736933	G/A	0.32	0.87	−0.05	−0.29 to 0.19	0.660	0.963	−0.02	−0.34 to 0.29	0.894	0.974	−0.20	−0.72 to 0.33	0.462	0.945
		rs10519567	26738703	G/A	0.30	0.73	−0.05	−0.30 to 0.19	0.684	0.963	−0.05	−0.36 to 0.27	0.772	0.974	−0.12	−0.68 to 0.44	0.681	0.993
		rs8024864	26751958	A/G	0.29	0.86	−0.05	−0.29 to 0.20	0.720	0.965	−0.03	−0.34 to 0.28	0.850	0.974	−0.15	−0.72 to 0.43	0.624	0.993
		rs80184385	26752620	A/G	0.07	0.04	0.13	−0.27 to 0.53	0.525	0.960	0.18	−0.30 to 0.66	0.462	0.957	0.04	−1.23 to 1.30	0.956	0.993
		rs8042817	26758205	C/T	0.26	1	−0.02	−0.27 to 0.23	0.887	0.986	−0.06	−0.37 to 0.25	0.708	0.974	0.13	−0.50 to 0.75	0.695	0.993
		rs3212335	26766994	G/A	0.37	0.35	−0.03	−0.27 to 0.21	0.803	0.973	−0.06	−0.39 to 0.26	0.699	0.974	0.02	−0.46 to 0.50	0.942	0.993
		rs8179186	26772389	G/A	0.37	0.28	0.01	−0.24 to 0.25	0.962	0.992	−0.11	−0.44 to 0.22	0.501	0.957	0.28	−0.21 to 0.77	0.269	0.941

(Continued)

Table S2. *(Continued)*

							Additive model				Dominant model				Recessive model			
**Gene**	**Chr**	**SNP**	**Position**	**A1/A2**	**MAF**	**HWE *P***	***β***	**95% CI**	***P***	***Q***	***β***	**95% CI**	***P***	***Q***	***β***	**95% CI**	***P***	***Q***
GABRD	1	rs3128320	2021579	G/A	0.07	0.57	0.01	−0.44 to 0.45	0.979	0.996	0.01	−0.46 to 0.48	0.964	0.994	−0.09	−2.32 to 2.13	0.937	0.993
		rs3128322	2022997	A/G	0.24	0.42	−0.04	−0.29 to 0.21	0.759	0.965	0.00	−0.31 to 0.32	0.984	0.994	−0.25	−0.86 to 0.37	0.428	0.945
		rs3128323	2023641	A/G	0.24	0.43	−0.03	−0.28 to 0.23	0.842	0.978	0.03	−0.29 to 0.34	0.876	0.974	−0.25	−0.86 to 0.37	0.428	0.945
		rs28574670	2027822	G/A	0.24	0.24	−0.08	−0.32 to 0.17	0.535	0.960	−0.04	−0.35 to 0.28	0.816	0.974	−0.32	−0.91 to 0.27	0.291	0.941
		rs28431879	2029969	C/T	0.32	0.74	−0.05	−0.29 to 0.19	0.658	0.963	−0.12	−0.43 to 0.19	0.443	0.957	0.09	−0.44 to 0.62	0.746	0.993
GABRE	X	rs12011671	151956493	T/C	0.50	0.85	−0.01	−0.26 to 0.24	0.938	0.986								
		rs2256882	151961350	G/A	0.35	0.02	0.04	−0.20 to 0.27	0.768	0.965								
		rs2256756	151962868	G/A	0.43	0.85	0.10	−0.14 to 0.35	0.419	0.960								
		rs1894367	151963790	A/G	0.48	0.45	0.07	−0.17 to 0.31	0.558	0.960								
		rs2266854	151965750	C/T	0.49	0.85	−0.03	−0.28 to 0.22	0.800	0.973								
		rs1139916	151969707	C/A	0.09	0.60	−0.16	−0.63 to 0.30	0.490	0.960								
		rs56192501	151971934	T/C	0.16	0.52	0.06	−0.29 to 0.40	0.740	0.965								
		rs2266856	151973060	C/T	0.21	0.06	−0.13	−0.46 to 0.20	0.439	0.960								
		rs2266858	151973503	T/A	0.38	0.56	−0.09	−0.34 to 0.16	0.484	0.960								
		rs2266859	151973735	A/G	0.47	0.45	−0.22	−0.46 to 0.02	0.068	0.749								
		rs5925077	151974325	C/T	0.31	0.67	0.14	−0.12 to 0.40	0.296	0.960								
GABRG1	4	rs993677	46053461	T/C	0.19	0.64	−0.10	−0.38 to 0.18	0.486	0.960	−0.15	−0.48 to 0.18	0.367	0.957	0.06	−0.71 to 0.84	0.874	0.993
		rs1603614	46072328	A/C	0.26	0.35	0.06	−0.19 to 0.30	0.650	0.963	0.02	−0.29 to 0.34	0.881	0.974	0.24	−0.34 to 0.82	0.424	0.945
		rs1248551	46072662	C/T	0.19	0.81	−0.12	−0.40 to 0.17	0.422	0.960	−0.16	−0.50 to 0.17	0.342	0.957	0.01	−0.82 to 0.83	0.988	0.994
		rs11945555	46088455	C/A	0.26	0.35	0.06	−0.19 to 0.30	0.650	0.963	0.02	−0.29 to 0.34	0.881	0.974	0.24	−0.34 to 0.82	0.424	0.945
		rs11736752	46089240	C/A	0.26	0.26	0.02	−0.22 to 0.27	0.853	0.979	−0.03	−0.35 to 0.29	0.844	0.974	0.22	−0.34 to 0.79	0.436	0.945
		rs1353640	46104826	A/C	0.33	0.20	0.07	−0.16 to 0.30	0.539	0.960	0.17	−0.14 to 0.49	0.278	0.957	−0.08	−0.55 to 0.38	0.732	0.993
		rs16859088	46106709	A/G	0.06	1	0.09	−0.39 to 0.56	0.721	0.965	0.09	−0.39 to 0.56	0.721	0.974	NA	NA	NA	NA
		rs12511372	46114415	A/G	0.27	0.20	0.00	−0.24 to 0.24	0.990	0.996	−0.08	−0.39 to 0.24	0.634	0.974	0.23	−0.31 to 0.77	0.401	0.945
		rs10938426	46116218	A/G	0.27	0.20	0.00	−0.24 to 0.24	0.990	0.996	−0.08	−0.39 to 0.24	0.634	0.974	0.23	−0.31 to 0.77	0.401	0.945
GABRG2	5	rs11742028	162070044	T/C	0.25	0.13	0.06	−0.21 to 0.33	0.654	0.963	−0.01	−0.32 to 0.30	0.964	0.994	0.56	−0.21 to 1.33	0.158	0.933
		rs17060039	162071539	T/C	0.31	0.73	−0.21	−0.45 to 0.02	0.077	0.749	−0.23	−0.54 to 0.08	0.152	0.841	−0.40	−0.91 to 0.12	0.131	0.933
		rs209351	162084848	C/A	0.09	1	−0.01	−0.40 to 0.37	0.950	0.986	−0.01	−0.41 to 0.39	0.971	0.994	−0.17	−2.34 to 2.00	0.876	0.993
		rs11135176	162095550	C/T	0.30	0.61	−0.24	−0.47 to 0.00	0.047	0.677	−0.27	−0.58 to 0.04	0.086	0.633	−0.40	−0.91 to 0.12	0.132	0.933
		rs2910223	162096717	A/G	0.36	0.75	0.05	−0.17 to 0.28	0.651	0.963	0.08	−0.23 to 0.40	0.608	0.974	0.04	−0.41 to 0.49	0.864	0.993
		rs211037	162101274	T/C	0.47	0.31	−0.13	−0.34 to 0.08	0.229	0.960	−0.31	−0.65 to 0.03	0.072	0.597	−0.02	−0.39 to 0.34	0.901	0.993
		rs211036	162102363	C/T	0.14	1	−0.06	−0.38 to 0.26	0.714	0.965	−0.07	−0.42 to 0.29	0.721	0.974	−0.10	−1.35 to 1.15	0.873	0.993
		rs74808690	162107787	C/A	0.06	0.51	0.13	−0.33 to 0.59	0.578	0.960	0.06	−0.43 to 0.55	0.823	0.974	1.81	−0.33 to 3.95	0.100	0.933
		rs210988	162108129	C/T	0.31	0.40	−0.01	−0.25 to 0.24	0.948	0.986	−0.09	−0.40 to 0.22	0.556	0.974	0.26	−0.30 to 0.82	0.360	0.941
		rs79780075	162144907	C/A	0.09	0.66	0.22	−0.16 to 0.60	0.259	0.960	0.31	−0.11 to 0.72	0.146	0.841	−0.64	−2.18 to 0.90	0.416	0.945

(Continued)

Table S2. *(Continued)*

							Additive model				Dominant model				Recessive model			
**Gene**	**Chr**	**SNP**	**Position**	**A1/A2**	**MAF**	**HWE *P***	***β***	**95% CI**	***P***	***Q***	***β***	**95% CI**	***P***	***Q***	***β***	**95% CI**	***P***	***Q***
GABRG3	15	rs2288694	26971599	C/T	0.20	0.50	0.19	−0.08 to 0.46	0.165	0.904	0.20	−0.12 to 0.53	0.223	0.913	0.39	−0.34 to 1.12	0.298	0.941
		rs2376481	26975566	T/C	0.48	0.56	0.08	−0.14 to 0.31	0.476	0.960	0.10	−0.25 to 0.45	0.581	0.974	0.12	−0.26 to 0.49	0.545	0.976
		rs7178872	26977835	G/A	0.13	0.54	0.05	−0.29 to 0.39	0.790	0.970	0.09	−0.27 to 0.45	0.621	0.974	−0.71	−2.25 to 0.82	0.364	0.941
		rs6606906	26980400	A/G	0.13	0.75	0.06	−0.29 to 0.40	0.739	0.965	0.11	−0.26 to 0.47	0.569	0.974	−0.72	−2.26 to 0.82	0.360	0.941
		rs1432133	26983199	C/T	0.13	0.74	0.08	−0.27 to 0.43	0.650	0.963	0.13	−0.24 to 0.50	0.484	0.957	−0.72	−2.26 to 0.82	0.360	0.941
		rs1432132	26983257	C/T	0.13	0.74	0.08	−0.27 to 0.43	0.650	0.963	0.13	−0.24 to 0.50	0.484	0.957	−0.72	−2.26 to 0.82	0.360	0.941
		rs12900861	26984852	C/T	0.13	0.74	0.08	−0.27 to 0.43	0.650	0.963	0.13	−0.24 to 0.50	0.484	0.957	−0.72	−2.26 to 0.82	0.360	0.941
		rs59299812	26985589	G/A	0.13	0.75	0.16	−0.16 to 0.48	0.328	0.960	0.19	−0.17 to 0.55	0.306	0.957	0.14	−0.94 to 1.22	0.798	0.993
		rs141832056	27008600	C/T	0.06	1	−0.18	−0.66 to 0.31	0.478	0.960	−0.18	−0.66 to 0.31	0.478	0.957	NA	NA	NA	NA
		rs6606855	27019010	G/A	0.27	0.85	0.05	−0.20 to 0.30	0.702	0.965	0.03	−0.28 to 0.35	0.833	0.974	0.16	−0.44 to 0.75	0.604	0.993
		rs2195815	27019873	G/A	0.34	0.26	−0.07	−0.29 to 0.15	0.548	0.960	0.02	−0.29 to 0.33	0.907	0.974	−0.33	−0.79 to 0.13	0.158	0.933
		rs61998080	27028312	G/A	0.12	0.72	−0.15	−0.49 to 0.19	0.391	0.960	−0.04	−0.42 to 0.34	0.826	0.974	−1.54	−2.77 to −0.31	0.015	0.933
		rs7167986	27030089	A/G	0.31	0.13	0.06	−0.20 to 0.31	0.653	0.963	0.14	−0.18 to 0.46	0.385	0.957	−0.18	−0.77 to 0.42	0.558	0.980
		rs35679219	27032888	C/T	0.19	0.24	0.01	−0.25 to 0.28	0.913	0.986	0.13	−0.19 to 0.46	0.426	0.957	−0.50	−1.20 to 0.19	0.160	0.933
		rs10519582	27033867	C/T	0.12	0.74	−0.13	−0.46 to 0.21	0.465	0.960	−0.02	−0.39 to 0.35	0.929	0.988	−1.54	−2.77 to −0.31	0.015	0.933
		rs56309634	27036144	A/G	0.22	0.09	−0.03	−0.28 to 0.22	0.820	0.975	0.07	−0.25 to 0.39	0.661	0.974	−0.44	−1.06 to 0.17	0.160	0.933
		rs79108611	27040978	C/T	0.31	0.40	0.01	−0.24 to 0.26	0.946	0.986	0.04	−0.28 to 0.35	0.824	0.974	−0.07	−0.65 to 0.50	0.802	0.993
		rs8031551	27042618	A/C	0.14	1	0.16	−0.16 to 0.48	0.331	0.960	0.19	−0.16 to 0.54	0.299	0.957	0.05	−1.19 to 1.3	0.932	0.993
		rs78295383	27043518	A/G	0.07	1	0.30	−0.13 to 0.73	0.171	0.913	0.33	−0.13 to 0.78	0.160	0.841	0.23	−1.92 to 2.38	0.834	0.993
		rs11263701	27045491	G/A	0.15	0.17	0.02	−0.27 to 0.31	0.910	0.986	0.09	−0.26 to 0.44	0.631	0.974	−0.35	−1.17 to 0.48	0.414	0.945
		rs12913876	27046059	G/A	0.16	0.42	−0.37	−0.68 to −0.06	0.019	0.468	−0.38	−0.72 to −0.05	0.026	0.482	−0.75	−2.00 to 0.50	0.241	0.941
		rs1030617	27048606	A/G	0.25	0.70	−0.21	−0.47 to 0.05	0.117	0.767	−0.26	−0.57 to 0.05	0.102	0.715	−0.20	−0.89 to 0.50	0.582	0.993
		rs1030616	27049002	G/A	0.20	0.83	−0.26	−0.53 to 0.02	0.067	0.749	−0.32	−0.64 to 0.00	0.048	0.497	−0.18	−1.01 to 0.65	0.676	0.993
		rs17647933	27049580	C/T	0.14	0.21	−0.49	−0.82 to −0.15	0.005	0.347	−0.49	−0.83 to −0.14	0.006	0.246	−1.09	−3.25 to 1.07	0.323	0.941
		rs12440850	27050228	A/G	0.11	0.25	−0.06	−0.40 to 0.28	0.750	0.965	−0.08	−0.47 to 0.32	0.700	0.974	0.02	−1.07 to 1.11	0.969	0.993
		rs891800	27050331	C/T	0.30	1	−0.21	−0.46 to 0.03	0.086	0.749	−0.32	−0.63 to 0.00	0.049	0.497	−0.13	−0.68 to 0.43	0.658	0.993
		rs12148267	27052103	G/A	0.15	1	0.16	−0.14 to 0.47	0.295	0.960	0.14	−0.20 to 0.48	0.422	0.957	0.64	−0.44 to 1.72	0.246	0.941
		rs17647963	27054578	G/A	0.16	0.10	−0.30	−0.58 to −0.02	0.037	0.659	−0.48	−0.82 to −0.14	0.006	0.246	0.16	−0.62 to 0.94	0.685	0.993
		rs1017363	27055226	C/T	0.28	0.05	−0.13	−0.39 to 0.14	0.359	0.960	−0.08	−0.40 to 0.23	0.605	0.974	−0.49	−1.22 to 0.24	0.192	0.941
		rs13379545	27055769	G/A	0.30	0.61	−0.07	−0.32 to 0.17	0.567	0.960	−0.01	−0.32 to 0.31	0.965	0.994	−0.37	−0.95 to 0.20	0.206	0.941
		rs57254597	27056047	C/T	0.14	1	0.07	−0.26 to 0.39	0.689	0.963	0.03	−0.32 to 0.39	0.851	0.974	0.56	−0.69 to 1.80	0.381	0.945
		rs77702559	27063782	A/G	0.18	0.81	0.04	−0.24 to 0.32	0.779	0.965	0.03	−0.30 to 0.36	0.866	0.974	0.17	−0.65 to 1.00	0.687	0.993
		rs12050742	27064847	G/A	0.45	0.56	0.12	−0.09 to 0.34	0.271	0.960	0.09	−0.24 to 0.43	0.583	0.974	0.26	−0.13 to 0.64	0.189	0.941
		rs6606868	27073785	T/C	0.15	0.58	−0.15	−0.47 to 0.17	0.359	0.960	−0.19	−0.54 to 0.15	0.277	0.957	0.21	−1.05 to 1.47	0.741	0.993
		rs7179575	27078052	C/T	0.09	1	0.03	−0.37 to 0.43	0.890	0.986	0.00	−0.42 to 0.42	0.993	0.997	0.88	−1.28 to 3.03	0.428	0.945
		rs1368023	27078928	A/G	0.09	1	−0.31	−0.69 to 0.08	0.118	0.767	−0.37	−0.77 to 0.03	0.074	0.600	0.84	−1.31 to 3.00	0.443	0.945

(Continued)

Table S2. *(Continued)*

							Additive model				Dominant model				Recessive model			
**Gene**	**Chr**	**SNP**	**Position**	**A1/A2**	**MAF**	**HWE *P***	***β***	**95% CI**	***P***	***Q***	***β***	**95% CI**	***P***	***Q***	***β***	**95% CI**	***P***	***Q***
GABRG3	15	rs4887525	27084191	T/C	0.06	0.47	0.13	−0.34 to 0.59	0.594	0.960	0.17	−0.32 to 0.67	0.497	0.957	−0.51	−2.66 to 1.64	0.642	0.993
		rs4479202	27116968	C/A	0.10	0.39	0.01	−0.35 to 0.36	0.977	0.996	−0.01	−0.41 to 0.40	0.967	0.994	0.14	−1.10 to 1.39	0.820	0.993
		rs8038005	27123330	A/G	0.49	0.56	0.19	−0.04 to 0.41	0.104	0.759	0.35	−0.01 to 0.71	0.056	0.512	0.14	−0.23 to 0.50	0.467	0.945
		rs208176	27133683	C/A	0.14	0.38	−0.44	−0.76 to −0.12	0.007	0.377	−0.45	−0.79 to −0.11	0.010	0.306	−0.95	−2.48 to 0.58	0.227	0.941
		rs2376837	27154342	C/T	0.11	1	−0.07	−0.43 to 0.29	0.705	0.965	−0.04	−0.43 to 0.34	0.831	0.974	−0.62	−2.17 to 0.93	0.436	0.945
		rs7402147	27164848	G/A	0.31	0.87	−0.24	−0.48 to −0.01	0.045	0.673	−0.22	−0.53 to 0.09	0.173	0.844	−0.59	−1.13 to −0.05	0.032	0.933
		rs17671946	27179855	C/T	0.36	0.06	−0.10	−0.35 to 0.14	0.410	0.960	−0.06	−0.38 to 0.26	0.727	0.974	−0.31	−0.83 to 0.21	0.242	0.941
		rs208124	27183746	A/G	0.34	0.42	0.15	−0.08 to 0.39	0.208	0.960	0.10	−0.21 to 0.42	0.519	0.965	0.44	−0.08 to 0.95	0.098	0.933
		rs4887528	27209334	C/T	0.34	0.52	0.03	−0.19 to 0.26	0.766	0.965	0.07	−0.24 to 0.38	0.668	0.974	−0.01	−0.48 to 0.47	0.974	0.993
		rs6422904	27211322	G/A	0.10	0.42	0.12	−0.23 to 0.47	0.494	0.960	0.12	−0.28 to 0.52	0.559	0.974	0.37	−0.88 to 1.62	0.561	0.980
		rs35770807	27214355	G/A	0.44	0.66	−0.09	−0.31 to 0.12	0.403	0.960	−0.17	−0.50 to 0.16	0.322	0.957	−0.07	−0.45 to 0.32	0.740	0.993
		rs7173587	27219705	C/T	0.09	0.37	−0.63	−1.03 to −0.23	0.002	0.269	−0.63	−1.03 to −0.23	0.002	0.201	NA	NA	NA	NA
		rs17648549	27219955	G/T	0.20	0.65	0.32	0.06 to 0.59	0.018	0.468	0.44	0.12 to 0.76	0.008	0.276	0.17	−0.56 to 0.91	0.648	0.993
		rs34755423	27223296	G/A	0.17	1	−0.17	−0.46 to 0.12	0.255	0.960	−0.21	−0.55 to 0.12	0.211	0.913	−0.06	−1.03 to 0.91	0.903	0.993
		rs12591221	27223330	T/C	0.05	0.41	−0.11	−0.60 to 0.37	0.649	0.963	−0.20	−0.72 to 0.32	0.457	0.957	1.14	−1.00 to 3.28	0.299	0.941
		rs1454665	27224489	A/G	0.34	0.42	−0.07	−0.32 to 0.17	0.545	0.960	−0.13	−0.45 to 0.19	0.428	0.957	0.00	−0.52 to 0.52	0.994	0.995
		rs8026901	27226495	A/G	0.50	0.39	0.15	−0.07 to 0.38	0.185	0.948	0.37	0.00 to 0.73	0.049	0.497	0.03	−0.34 to 0.40	0.864	0.993
		rs74248639	27227792	A/G	0.09	0.66	−0.22	−0.60 to 0.16	0.254	0.960	−0.30	−0.72 to 0.11	0.149	0.841	0.56	−0.97 to 2.09	0.473	0.945
		rs79617243	27235219	T/C	0.09	0.63	−0.17	−0.56 to 0.21	0.379	0.960	−0.12	−0.54 to 0.30	0.578	0.974	−1.14	−2.66 to 0.37	0.140	0.933
		rs7164819	27236634	C/T	0.10	0.70	−0.10	−0.48 to 0.27	0.594	0.960	−0.10	−0.49 to 0.29	0.599	0.974	−0.18	−2.34 to 1.98	0.870	0.993
		rs28584617	27236826	A/G	0.48	0.77	−0.08	−0.30 to 0.15	0.504	0.960	0.00	−0.36 to 0.36	0.985	0.994	−0.21	−0.57 to 0.16	0.278	0.941
		rs11857892	27246758	T/C	0.38	0.54	−0.20	−0.44 to 0.03	0.087	0.749	−0.12	−0.44 to 0.21	0.492	0.957	−0.54	−0.99 to −0.10	0.018	0.933
		rs112462623	27249405	G/A	0.07	0.57	0.32	−0.12 to 0.77	0.157	0.869	0.35	−0.12 to 0.82	0.150	0.841	0.34	−1.82 to 2.50	0.761	0.993
		rs28616093	27251336	C/T	0.16	0.58	−0.18	−0.49 to 0.13	0.266	0.960	−0.21	−0.54 to 0.13	0.238	0.913	−0.07	−1.31 to 1.18	0.914	0.993
		rs75435047	27256663	C/T	0.12	0.73	0.35	0.02 to 0.69	0.039	0.659	0.40	0.03 to 0.77	0.037	0.485	0.43	−0.82 to 1.69	0.500	0.951
		rs7174776	27261389	A/G	0.42	1	−0.11	−0.33 to 0.12	0.350	0.960	−0.12	−0.45 to 0.22	0.497	0.957	−0.18	−0.59 to 0.23	0.386	0.945
		rs28558146	27261910	T/C	0.07	0.60	−0.12	−0.56 to 0.32	0.596	0.960	−0.12	−0.56 to 0.32	0.596	0.974	NA	NA	NA	NA
		rs17137734	27262626	C/T	0.07	0.60	−0.12	−0.56 to 0.32	0.596	0.960	−0.12	−0.56 to 0.32	0.596	0.974	NA	NA	NA	NA
		rs75436954	27264028	G/A	0.12	0.49	0.34	0.01 to 0.67	0.042	0.659	0.42	0.05 to 0.79	0.029	0.485	0.23	−0.86 to 1.33	0.678	0.993
		rs1869599	27265642	C/T	0.19	0.36	0.30	0.03 to 0.57	0.029	0.603	0.38	0.06 to 0.70	0.023	0.482	0.31	−0.43 to 1.06	0.412	0.945
		rs7177867	27273282	T/C	0.08	0.61	−0.19	−0.63 to 0.25	0.397	0.960	−0.19	−0.63 to 0.25	0.397	0.957	NA	NA	NA	NA
		rs2061051	27275664	G/A	0.40	0.45	0.02	−0.20 to 0.24	0.881	0.986	−0.03	−0.35 to 0.29	0.857	0.974	0.11	−0.30 to 0.52	0.610	0.993
		rs13380002	27285104	T/C	0.08	0.62	−0.26	−0.69 to 0.17	0.233	0.960	−0.26	−0.69 to 0.17	0.233	0.913	NA	NA	NA	NA
		rs6606897	27294162	C/A	0.27	0.36	−0.03	−0.28 to 0.23	0.850	0.979	−0.10	−0.42 to 0.21	0.512	0.957	0.31	−0.35 to 0.97	0.363	0.941
		rs7495025	27311812	G/A	0.29	0.72	−0.04	−0.29 to 0.21	0.763	0.965	−0.08	−0.40 to 0.23	0.594	0.974	0.09	−0.51 to 0.68	0.770	0.993
		rs77628044	27312468	C/A	0.14	1	−0.12	−0.44 to 0.19	0.443	0.960	−0.17	−0.52 to 0.19	0.362	0.957	0.08	−1.00 to 1.17	0.882	0.993

(Continued)

Table S2. *(Continued)*

Gene	Chr	SNP	Position	A1/A2	MAF	HWE P	Additive model β	Additive model 95% CI	Additive model P	Additive model Q	Dominant model β	Dominant model 95% CI	Dominant model P	Dominant model Q	Recessive model β	Recessive model 95% CI	Recessive model P	Recessive model Q
GABRG3	15	rs78159599	27312891	A/G	0.14	1	−0.12	−0.44 to 0.19	0.443	0.960	−0.17	−0.52 to 0.19	0.362	0.957	0.08	−1.00 to 1.17	0.882	0.993
		rs79790387	27313457	A/G	0.14	1	−0.12	−0.44 to 0.19	0.443	0.960	−0.17	−0.52 to 0.19	0.362	0.957	0.08	−1.00 to 1.17	0.882	0.993
		rs10438508	27314663	G/A	0.14	1	−0.12	−0.44 to 0.19	0.443	0.960	−0.17	−0.52 to 0.19	0.362	0.957	0.08	−1.00 to 1.17	0.882	0.993
		rs58606326	27315440	C/A	0.15	1	−0.13	−0.45 to 0.19	0.419	0.960	−0.17	−0.53 to 0.18	0.340	0.957	0.08	−1.00 to 1.17	0.882	0.993
		rs77721650	27316564	G/A	0.13	0.52	−0.12	−0.45 to 0.20	0.460	0.960	−0.17	−0.53 to 0.20	0.374	0.957	0.08	−1.00 to 1.17	0.882	0.993
		rs77291331	27318498	G/A	0.12	0.49	−0.10	−0.43 to 0.23	0.543	0.960	−0.14	−0.52 to 0.23	0.455	0.957	0.08	−1.00 to 1.17	0.882	0.993
		rs12914579	27319828	A/G	0.12	0.72	−0.08	−0.42 to 0.27	0.661	0.963	−0.08	−0.46 to 0.30	0.665	0.974	−0.11	−1.37 to 1.15	0.859	0.993
		rs79260395	27322691	A/G	0.08	1	−0.05	−0.47 to 0.37	0.818	0.975	−0.05	−0.49 to 0.39	0.834	0.974	−0.18	−2.34 to 1.98	0.870	0.993
		rs77479239	27323692	C/T	0.06	1	−0.08	−0.56 to 0.41	0.759	0.965	−0.08	−0.56 to 0.41	0.759	0.974	NA	NA	NA	NA
		rs28491109	27328145	G/A	0.09	0.63	−0.21	−0.60 to 0.17	0.280	0.960	−0.23	−0.66 to 0.19	0.284	0.957	−0.32	−1.86 to 1.21	0.681	0.993
		rs28564251	27333958	G/A	0.40	0.77	−0.29	−0.51 to −0.07	0.011	0.391	−0.45	−0.77 to −0.13	0.006	0.246	−0.26	−0.68 to 0.16	0.231	0.941
		rs67338000	27339693	G/A	0.49	0.89	−0.17	−0.39 to 0.06	0.146	0.855	−0.27	−0.63 to 0.09	0.142	0.841	−0.16	−0.53 to 0.20	0.379	0.945
		rs145560226	27342364	G/A	0.09	0.38	0.01	−0.38 to 0.41	0.944	0.986	0.01	−0.38 to 0.41	0.944	0.994	NA	NA	NA	NA
		rs149919840	27344970	C/T	0.20	1	−0.08	−0.36 to 0.20	0.574	0.960	−0.08	−0.41 to 0.25	0.646	0.974	−0.19	−0.97 to 0.58	0.626	0.993
		rs28671569	27352781	G/A	0.41	1	0.09	−0.14 to 0.32	0.436	0.960	0.11	−0.22 to 0.44	0.531	0.974	0.14	−0.28 to 0.55	0.517	0.965
		rs9672616	27357223	T/C	0.21	1	−0.04	−0.32 to 0.24	0.773	0.965	−0.02	−0.35 to 0.30	0.892	0.974	−0.19	−0.97 to 0.58	0.626	0.993
		rs9330519	27358707	A/G	0.19	0.64	−0.04	−0.32 to 0.24	0.785	0.968	−0.02	−0.35 to 0.31	0.907	0.974	−0.19	−0.97 to 0.58	0.626	0.993
		rs10083606	27363615	G/A	0.19	1	−0.06	−0.35 to 0.22	0.655	0.963	−0.04	−0.37 to 0.29	0.813	0.974	−0.30	−1.13 to 0.53	0.474	0.945
		rs10083616	27364025	G/A	0.19	1	−0.08	−0.36 to 0.21	0.593	0.960	−0.06	−0.39 to 0.27	0.734	0.974	−0.30	−1.13 to 0.53	0.474	0.945
		rs9708195	27370766	G/A	0.05	1	−0.07	−0.57 to 0.43	0.780	0.965	−0.07	−0.57 to 0.43	0.780	0.974	NA	NA	NA	NA
		rs28549123	27373335	A/G	0.34	1	−0.07	−0.30 to 0.16	0.544	0.960	−0.13	−0.45 to 0.18	0.411	0.957	0.00	−0.49 to 0.49	0.995	0.995
		rs62001350	27384143	C/T	0.23	0.10	0.05	−0.24 to 0.33	0.744	0.965	0.05	−0.27 to 0.37	0.750	0.974	0.06	−0.83 to 0.95	0.893	0.993
		rs9672753	27386740	T/C	0.34	1	−0.16	−0.39 to 0.07	0.178	0.930	−0.22	−0.53 to 0.09	0.168	0.844	−0.17	−0.65 to 0.32	0.495	0.951
		rs28816726	27393042	T/C	0.19	0.35	−0.18	−0.45 to 0.09	0.186	0.948	−0.23	−0.55 to 0.10	0.174	0.844	−0.21	−0.94 to 0.52	0.578	0.993
		rs55822510	27393628	G/A	0.10	0.70	−0.20	−0.58 to 0.17	0.284	0.960	−0.16	−0.55 to 0.23	0.424	0.957	−1.92	−4.05 to 0.22	0.080	0.933
		rs9635413	27395478	G/A	0.21	1	0.16	−0.11 to 0.44	0.245	0.960	0.15	−0.17 to 0.47	0.367	0.957	0.45	−0.33 to 1.23	0.261	0.941
		rs12440080	27400076	G/A	0.26	1	0.19	−0.06 to 0.45	0.131	0.814	0.23	−0.09 to 0.54	0.160	0.841	0.29	−0.33 to 0.92	0.355	0.941
		rs3924706	27414229	A/G	0.29	0.73	−0.12	−0.36 to 0.13	0.339	0.960	−0.19	−0.50 to 0.12	0.240	0.913	−0.02	−0.59 to 0.56	0.948	0.993
		rs71465230	27441743	C/T	0.06	0.51	0.20	−0.25 to 0.66	0.382	0.960	0.25	−0.23 to 0.74	0.312	0.957	−0.37	−2.56 to 1.82	0.739	0.993
		rs12906172	27461915	A/C	0.08	1	0.08	−0.33 to 0.48	0.713	0.965	0.10	−0.33 to 0.53	0.652	0.974	−0.37	−2.56 to 1.82	0.739	0.993
		rs74006954	27467498	G/A	0.08	1	0.12	−0.29 to 0.53	0.563	0.960	0.15	−0.28 to 0.58	0.501	0.957	−0.37	−2.56 to 1.82	0.739	0.993
		rs7170988	27468690	T/C	0.27	1	−0.11	−0.37 to 0.14	0.379	0.960	−0.15	−0.47 to 0.17	0.352	0.957	−0.10	−0.72 to 0.51	0.743	0.993
		rs7167588	27479150	G/A	0.38	0.06	−0.08	−0.32 to 0.17	0.550	0.960	0.00	−0.33 to 0.34	0.981	0.994	−0.32	−0.82 to 0.18	0.212	0.941
		rs34315951	27487554	A/G	0.07	0.60	−0.01	−0.44 to 0.42	0.963	0.992	0.00	−0.46 to 0.47	0.984	0.994	−0.37	−2.56 to 1.82	0.739	0.993
		rs34111854	27488003	G/A	0.07	0.57	−0.02	−0.45 to 0.42	0.947	0.986	0.00	−0.47 to 0.47	1.000	1.000	−0.37	−2.56 to 1.82	0.739	0.993
		rs12906479	27496163	T/C	0.34	0.75	0.00	−0.23 to 0.24	0.968	0.995	0.02	−0.29 to 0.34	0.896	0.974	−0.03	−0.53 to 0.47	0.903	0.993

(Continued)

Table S2. *(Continued)*

Gene	Chr	SNP	Position	A1/A2	MAF	HWE *P*	Additive model *β*	Additive 95% CI	Additive *P*	Additive *Q*	Dominant model *β*	Dominant 95% CI	Dominant *P*	Dominant *Q*	Recessive model *β*	Recessive 95% CI	Recessive *P*	Recessive *Q*
GABRG3	15	rs11074282	27496638	T/C	0.27	0.85	0.00	−0.25 to 0.25	0.997	0.997	0.05	−0.26 to 0.36	0.754	0.974	−0.20	−0.83 to 0.42	0.527	0.966
		rs34366812	27496660	A/G	0.14	1	−0.10	−0.42 to 0.21	0.531	0.960	−0.19	−0.54 to 0.16	0.295	0.957	0.61	−0.49 to 1.72	0.276	0.941
		rs12592749	27497255	T/C	0.48	0.89	−0.05	−0.27 to 0.17	0.640	0.963	−0.12	−0.47 to 0.23	0.509	0.957	−0.02	−0.39 to 0.35	0.930	0.993
		rs8027309	27501432	C/T	0.50	0.56	0.08	−0.15 to 0.30	0.507	0.960	0.13	−0.23 to 0.49	0.476	0.957	0.07	−0.30 to 0.43	0.718	0.993
		rs4778147	27507599	C/T	0.29	0.86	0.15	−0.09 to 0.39	0.222	0.960	0.20	−0.10 to 0.51	0.196	0.909	0.14	−0.42 to 0.70	0.623	0.993
		rs73369559	27508213	C/A	0.43	0.18	0.10	−0.13 to 0.33	0.407	0.960	0.11	−0.22 to 0.45	0.509	0.957	0.15	−0.28 to 0.58	0.492	0.951
		rs3101641	27524144	G/A	0.26	0.35	−0.07	−0.31 to 0.17	0.553	0.960	−0.17	−0.48 to 0.14	0.292	0.957	0.15	−0.41 to 0.71	0.608	0.993
		rs2303879	27527746	A/G	0.22	0.67	0.07	−0.19 to 0.33	0.587	0.960	−0.02	−0.34 to 0.30	0.894	0.974	0.62	−0.07 to 1.31	0.082	0.933
GABRQ	X	rs10482208	152642426	C/T	0.11	1	0.08	−0.31 to 0.47	0.679	0.963								
		rs3810650	152650860	A/G	0.33	0.54	0.00	−0.25 to 0.26	0.991	0.996								
		rs3810651	152652814	T/A	0.31	0.41	0.01	−0.25 to 0.27	0.952	0.986								
		rs5925199	152655640	T/C	0.32	0.30	0.02	−0.23 to 0.28	0.861	0.982								
		rs1129943	152657424	C/T	0.27	1	−0.01	−0.29 to 0.27	0.937	0.986								
		rs4828705	152659103	A/G	0.32	0.30	0.02	−0.23 to 0.28	0.861	0.982								

Variants on the X chromosome were excluded from the analyses for the dominant and recessive models. The regression coefficients (β) represent the effect size and direction with the major allele as the reference allele. Values in bold indicates significant results with $Q < 0.05$.

Chr, chromosome; SNP, single-nucleotide polymorphism; Position, chromosomal position in Genome Reference Consortium Human Build 38; A1, major allele; A2, minor allele; MAF, minor allele frequency; HWE, Hardy-Weinberg equilibrium exact test; β, regression coefficient; CI, confidence interval; Q, false discovery rate P; NA, not available.
